# 1.8 Billion Years of Detrital Zircon Recycling Calibrates a Refractory Part of Earth’s Sedimentary Cycle

**DOI:** 10.1371/journal.pone.0144727

**Published:** 2015-12-14

**Authors:** Thomas Hadlari, Graeme T. Swindles, Jennifer M. Galloway, Kimberley M. Bell, Kyle C. Sulphur, Larry M. Heaman, Luke P. Beranek, Karen M. Fallas

**Affiliations:** 1 Geological Survey of Canada, Calgary, Alberta, Canada; 2 University of Leeds, School of Geography, Leeds, United Kingdom; 3 University of Calgary, Department of Geoscience, Calgary, Alberta, Canada; 4 University of Alberta, Department of Earth and Atmospheric Sciences, Edmonton, Alberta, Canada; 5 Memorial University of Newfoundland, Department of Earth Sciences, St. John’s, Newfoundland, Canada; University of California Los Angeles, UNITED STATES

## Abstract

Detrital zircon studies are providing new insights on the evolution of sedimentary basins but the role of sedimentary recycling remains largely undefined. In a broad region of northwestern North America, this contribution traces the pathway of detrital zircon sand grains from Proterozoic sandstones through Phanerozoic strata and argues for multi-stage sedimentary recycling over more than a billion years. As a test of our hypothesis, integrated palynology and detrital zircon provenance provides clear evidence for erosion of Carboniferous strata in the northern Cordillera as a sediment source for Upper Cretaceous strata. Our results help to calibrate Earth's sedimentary cycle by showing that recycling dominates sedimentary provenance for the refractory mineral zircon.

## Introduction

**Fig 1:**
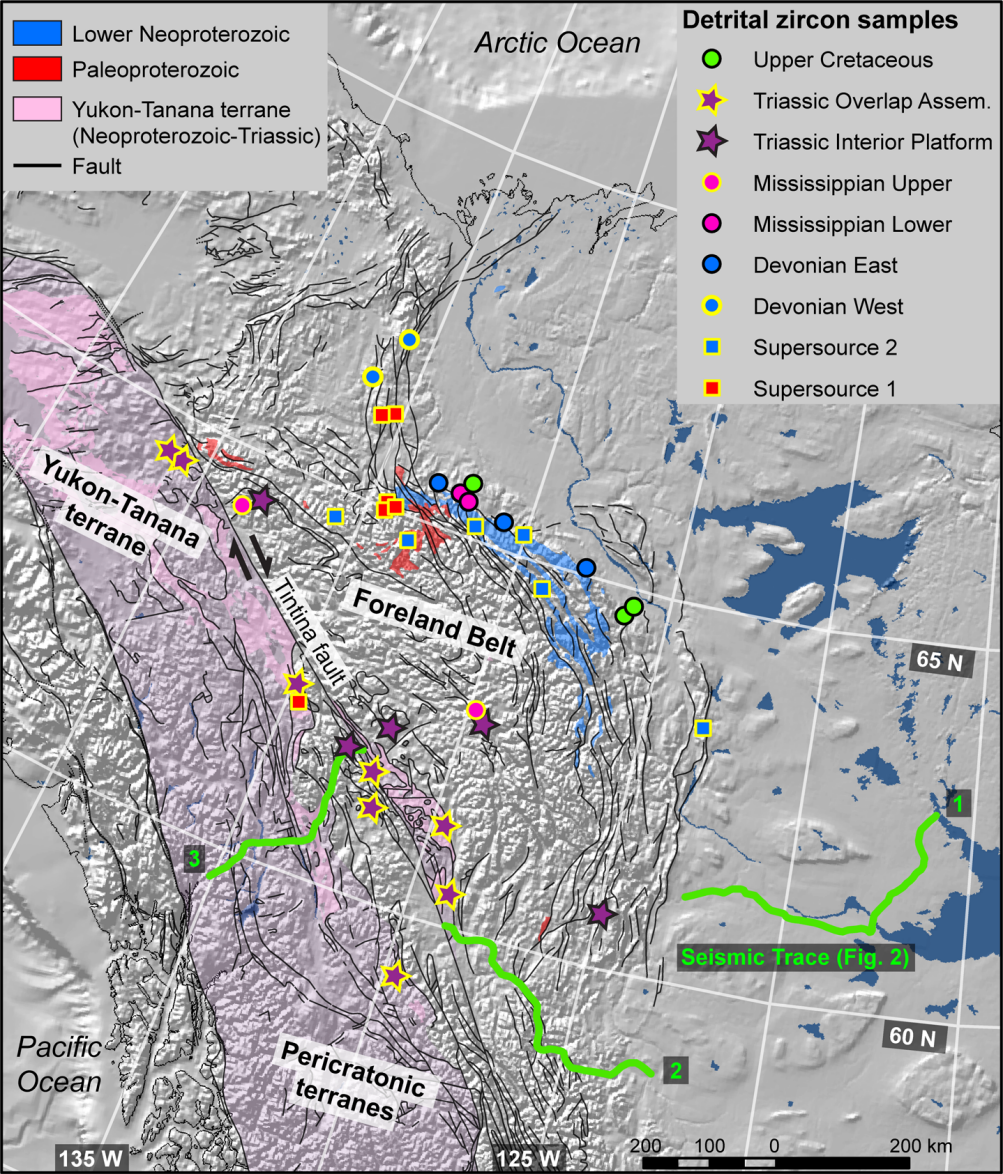
Location map. Detrital zircon sample locations within the northern Cordillera of North America. The pericratonic Yukon Tanana terrane was a Devonian arc built upon Laurentian (North American) basement [10]. The Mesozoic Interior platform is approximated by the Foreland Belt.

**Fig 2:**
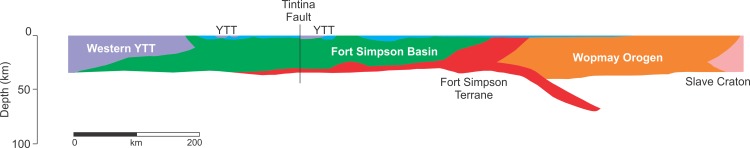
Seismic cross-section of the crust. Crustal-scale section across the North American Cordillera. Interpretations are based on reflection seismic data [11]. Undivided Proterozoic includes Paleo- to Neoproterozoic strata. The Paleoproterozoic Fort Simpson Basin formed after rifting and attenuation of Fort Simpson terrane crust at approximately 1.8 Ga, and was filled by continental slope and terrace deposits before 1.6 Ga. The western portion of greater Yukon-Tanana terrane (YTT) includes parts of other pericratonic terranes [10].

Mature framework geology and a ~1.8 billion year detrital zircon record from the northern Cordillera of North America provide exceptional context to explore the dynamics of sediment erosion and redeposition over deep geologic time (Table 1; Figs 1 and 2). An integration of bedrock geology, crustal-scale seismology, and detrital zircon U-Pb geochronology identifies two overarching reservoirs of detrital zircon in the form of Proterozoic sedimentary rocks with 2.0–1.8 Ga and 1.5–1.0 Ga detrital zircon signatures. Temporal statistical analysis is developed for correlation of time-windows within detrital zircon age spectra in order to identify older spectral signals within younger strata and to reconstruct recycling patterns through the Phanerozoic. This approach is applied in particular detail to Upper Cretaceous strata because a second proxy for sediment provenance is available in the form of recycled pollen and spores. If two different stratigraphic units have identical detrital zircon age spectra, then equivocal interpretations are that they had the same sediment sources or that one might have been eroded to source the other, but our integration of detrital palynomorph provenance allows for a clear interpretation of sedimentary recycling.

**Table 1:** Geological history and provenance.

Age	Basin-tectonic setting	Provenance
Upper Cretaceous	Foreland basin	Recycling of older rocks within the Cordillera [1]
Triassic	Overlap assemblage; Yukon-Tanana terrane and Interior Platform	Yukon-Tanana terrane, recycled Interior Platform ± Cordilleran basement [2,3]
Carboniferous	Back-arc basin to continental shelf	Recycled Paleozoic Platform ± Cordilleran basement [4]
Devonian	Distal Ellesmerian foreland basin	Accreted terranes in arctic regions ± Laurentian basins [4,5]
Cambrian and U. Neoproterozoic	Passive margin	Local recycling of Proterozoic sedimentary rocks [6,7]
L. Neoproterozoic	~1.0–0.7 Ga Rodinian continental interior	Distal Mesoproterozoic orogens [8]
Paleoproterozoic	~1.8–1.6 Ga margin	Laurentian craton [9]

Summary of the tectonic setting and provenance of major siliciclastic successions of the northern Cordillera [1–9]. Pre-1.8 Ga basement forms a thin highly attenuated layer at the base of the crust (see Fig 2).

### The philosophy of detrital zircon provenance

Geochronological studies of highly resistant detrital zircon grains are reshaping our view of how sediment moves through sedimentary basins and across continents in response to global tectonics [12]. The phenomenon of recycling is an established part of the sedimentary cycle, nonetheless, detrital zircon provenance is often attributed to nearly contemporaneous erosion of crystalline rocks and direct sediment transport. It is known, however, that sand-sized detrital zircon grains can be multiply recycled within sedimentary systems whilst retaining robust U-Pb crystallization ages as a record of their ultimate sources [13,14]. A logical extension of the recycling process is that after the original crystalline source rock has been eroded, it is those subsequent deposits that themselves become sources of detrital zircon leading to “sedimentary inheritance” of age spectra. It is therefore likely that over time the recycling process will expand the geographic distributions of detrital zircon grains with U-Pb age associations and distributed age probability patterns that are displaced from their ultimate sources.

## Methods

**Fig 3:**
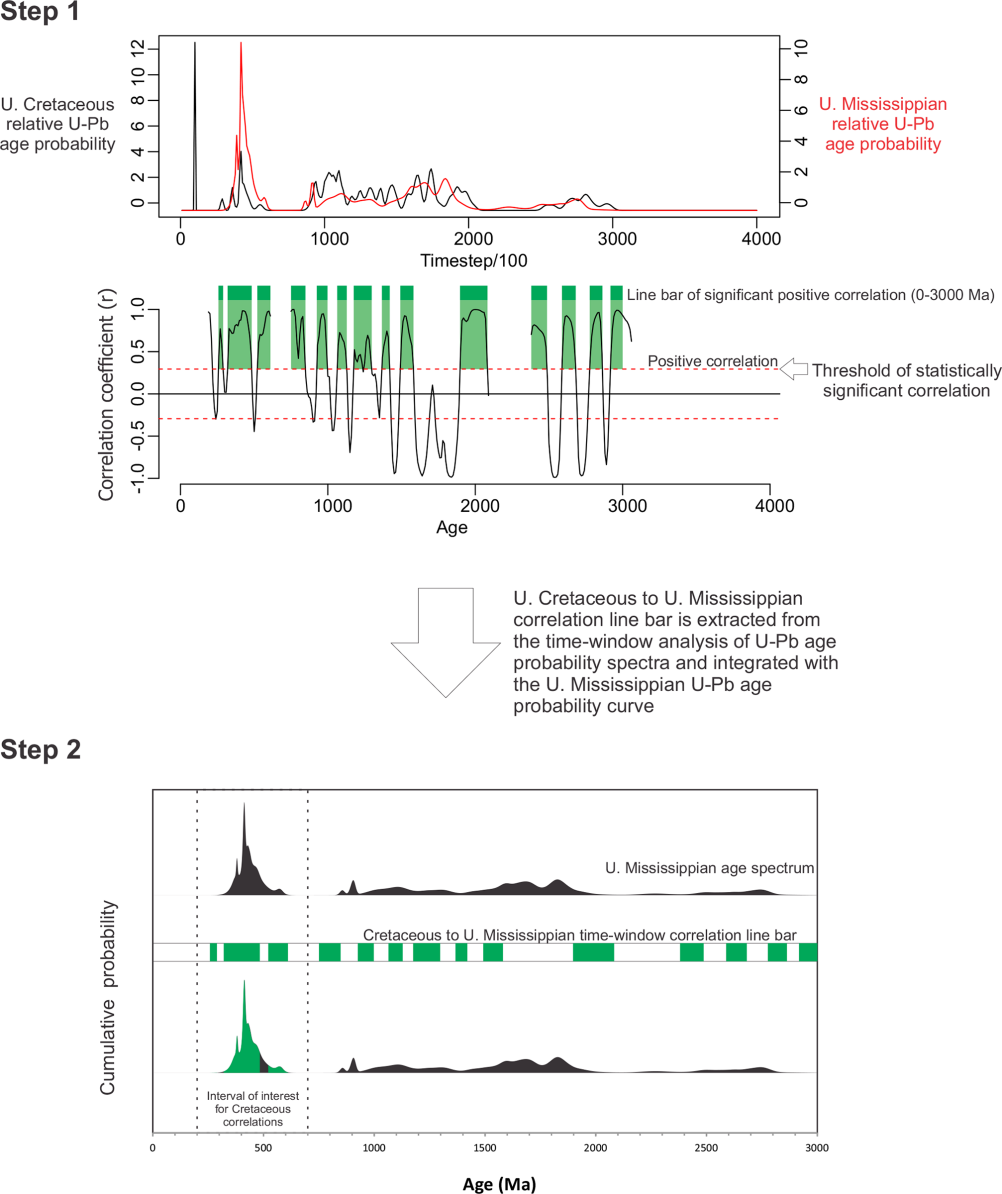
U-Pb age-probability distributions. Example of the workflow for converting temporal statistical correlations between two detrital zircon U-Pb cumulative probability age spectra (Step 1) to relative probability space under a single curve (Step 1). In Step 2, the portion of the U. Mississippian age spectrum that is coloured green correlates to the U. Cretaceous age spectrum.

**Fig 4:**
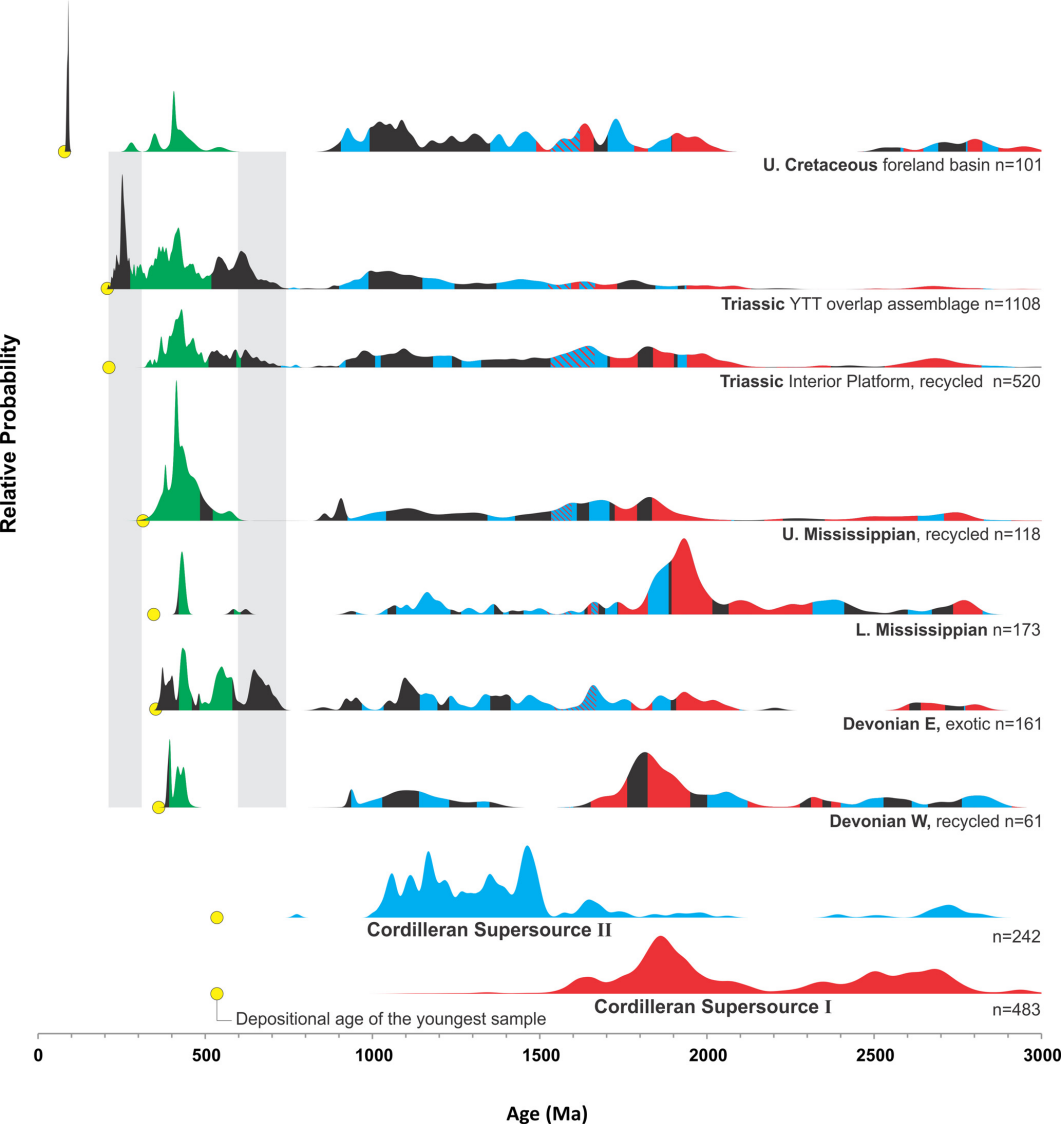
U-Pb age-probability distributions. Relative probability-age plots of detrital zircon U-Pb sample sets. Statistically significant time-window correlation between Cordilleran supersource spectra and Phanerozoic sample groups are indicated by respective red and blue fill under the Phanerozoic age-probability curves. An example time-window correlation between two samples is shown in Fig 3. Upper Cretaceous time-window correlations with older Phanerozoic groups are shown for the age interval 700–200 Ma.

No permits were required for the described study, which complied with all relevant regulations. Representative detrital zircon U-Pb datasets from the northern Cordillera were tabulated, filtered at 5% discordance, and are displayed using relative probability–age plots. New samples were processed and analysed using LA-MC-ICP-MS procedures at the University of Alberta. Simple comparative analysis of detrital zircon age probability spectra is aided by statistical time-series analysis. Probability values from the cumulative age plots include all error probabilities and were analysed in pairs (Fig 3, Step 1). We used bivariate running correlation analysis (Pearson r) which is a standard method to determine temporal statistical correlations between age probability distributions, but not applied to detrital zircon age data previously. A time window of 100 Ma was used. A Monte Carlo procedure for significance testing was applied. Each Phanerozoic sample set was tested for correlation to both Proterozoic “supersource” age spectra and to the Upper Cretaceous age spectrum (e.g., Fig 3). Key age intervals with statistically significant correlation are colour coded in Fig 4, and a workflow example is shown in Fig 3. Palynological samples were collected from well cuttings (Little Bear Formation) and outcrop (Trevor Formation), with processing and analysis at the Geological Survey of Canada. S1 Appendix contains supplemental geochronological (S1 and S2 Figs), statistical, and palynological methods and results.

## Results and Discussion

### Precambrian sedimentary rocks store detrital zircons

The history of detrital zircon recycling in the northern Cordillera starts after 1.9–1.84 Ga Wopmay orogenesis, when rifting led to a passive margin along northwestern Laurentia, sometimes called the ancestral North American craton, at approximately 1.8 Ga [11]. Sediment eroded from Paleoproterozoic orogens prograded from the cratonic interior over attenuated crust to form a continental slope and terrace [15]. Those continental margin deposits of the Fort Simpson Basin presently compose much of the crustal thickness below the Foreland Belt and eastern Yukon-Tanana terrane (Fig 2). The main reservoir of 2.0–1.8 Ga detrital zircon in the northern Cordillera is therefore Paleoproterozoic (meta-) sedimentary rocks [16] and any sediment eroded from this reservoir will provide recycled detrital zircon grains which were ultimately derived from the cratonic interior of Laurentia. In the Paleozoic, the Yukon-Tanana terrane was a Devonian arc built upon Laurentian basement [10] containing 2.0–1.8 Ga detrital zircons [17]. To characterize Cordilleran Supersource I we combine detrital zircon age spectra from Paleoproterozoic outcrops in the Foreland Belt [9,18], Cambrian derivatives [7], and Yukon-Tanana terrane basement [17] (Fig 4). Detrital zircon samples of lower Neoproterozoic strata older than 0.72 Ga cannot be correlated to an ultimate source in the region and were probably transported long distances during the Neoproterozoic [8]. Samples of lower Neoproterozoic sandstones and their Cambrian derivatives [6] are combined to characterize Supersource II, which is dominated by 1.5–1.0 Ga age probability (Fig 4). Sediment eroded from the Supersource II reservoir will provide recycled detrital zircon ultimately from a distant source region.

### Sedimentary recycling through the Phanerozoic

Detrital zircon provenance studies from the main siliciclastic successions of the northern Cordillera propose multiple stages of regional sedimentary recycling interspersed with influx of sediment from arcs coupled with exotic crustal sources (Table 1). Orogenic events result in detrital zircon populations derived from regional recycling as well as arcs as a function of basin geometry [1], which the Devonian and Triassic sample sets show in Fig 4. The recycled Devonian sample set is dominated by Precambrian ages with good correlation to the “Cordilleran supersource I”, with lesser fractions derived from Paleozoic arcs. Detrital zircons from the exotic Devonian sample set were derived from Paleozoic arcs and 550–700 Ma crust accreted to arctic Laurentian margins marking an influx of exotic sediment to the northern Cordilleran region [4–5]. The broad Supersource II correlations are likely due to the presence of Neoproterozoic strata distributed across northern Laurentia that quite likely had the same distant ultimate source as equivalent strata in the northern Cordillera [8]. The post-orogenic Upper Mississippian set records a period of sedimentary recycling [4] consistent with derivation, probably polycyclic, from Proterozoic “supersources” and lower Paleozoic arcs. Western Devonian, and any older, strata would be suitable proximate sources. The recycled suite of the Triassic Interior Platform contains age probabilities overlapping with all older strata [2], including the exotic 550–700 Ma fractions indicating relatively broad provenance. The Triassic overlap assemblage between the Interior Platform and Yukon-Tanana terrane is distinguished by lesser proportions of Precambrian ages and prominent Permo-Triassic age probabilities that record provenance from Yukon-Tanana terrane arc rocks [3]. Sedimentary provenance during the Upper Cretaceous was from the Cordillera west of the foreland basin, and the Proterozoic “supersource” signatures compose most of the detrital zircon age probability. In summary, the statistical representation is consistent with Phanerozoic provenance interpretations of multi-stage recycling [1–7]. The emerging pattern is that arc magmatism and accretion provide influx of new detrital zircon age fractions to the region, captured by contemporaneous deposition, which tend to be diluted by older fractions during subsequent stages of sedimentary reworking and recycling.

### Recycling of detrital zircons and palynomorphs during Cretaceous time

Upper Cretaceous strata preserved adjacent to the Foreland Belt were deposited in a foreland basin with Cordilleran provenance that readily explains near syn-depositional detrital zircons [1]. Starting with the proposition that detrital zircons within Upper Cretaceous strata are likely derived from erosion of older strata within the Cordillera [1], we assume that if sedimentary rocks are eroded, then the main components of the entire age spectrum will be recycled. The portion of the age spectrum greater than 1000 Ma could be inherited from erosion of almost any older strata and so to narrow down potential sources the younger portion of the age spectrum is subdivided into 750–600 Ma, 600–500 Ma, 500–350 Ma, and 350–200 Ma (Fig 4). The Upper Cretaceous spectrum has peaks ranging from 600–350 Ma that only overlap comprehensively with the Upper Mississippian spectrum, with the exception of a single ca. 281 Ma grain. It should be noted that the Devonian West sample set has a relatively low number of analyses (n = 61), and could be considered a possible sediment source. With this caveat, the Upper Cretaceous 600–350 Ma age probability is statistically equivalent to this interval in the Upper Mississippian age spectrum. The resulting deduction is that erosion of Upper Mississippian strata could provide all the detrital zircons necessary to produce the age probability of the Upper Cretaceous samples, except for the ca. 281 Ma grain.

**Fig 5:**
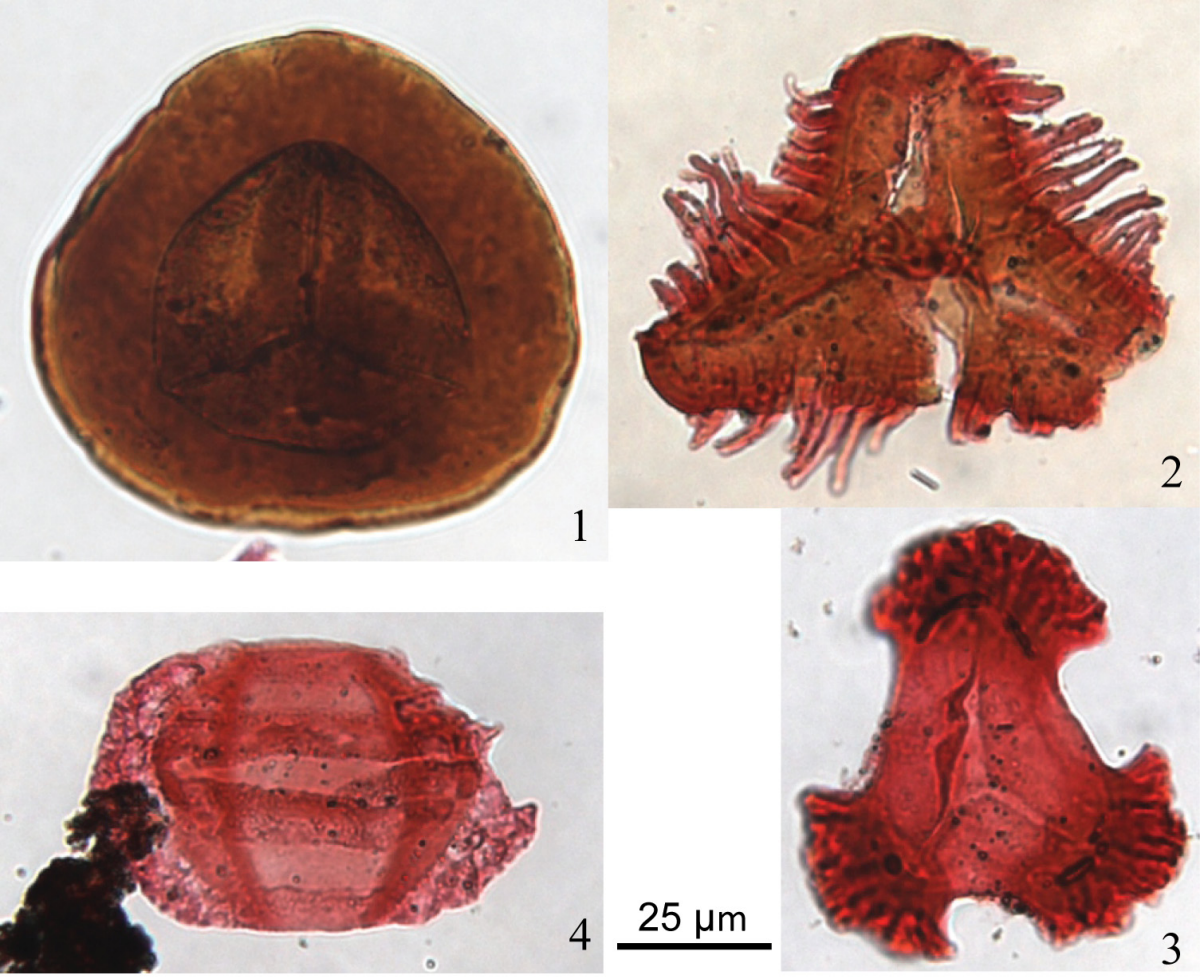
Palynomorph microphotographs. Recycled palynomorphs within Upper Cretaceous strata. Carboniferous spores are (1) *Densosporites* sp., (2) *Diatomozonotriletes* sp., and (3) *Triquitrites* sp. (Mississippian). The taeniate bisaccate pollen grain is Permian-Early Triassic (4).

Aside from crystallization ages and associations, detrital zircon U-Pb analysis does not provide diagnostic information to identify sedimentary sources, and certainly not the depositional age of candidates for sedimentary cannibalization. The same Upper Cretaceous formations that were sampled for detrital zircon have palynological assemblages comprising up to 23% recycled palynomorphs, and these are age diagnostic of the strata from whence they came (Fig 5). The recycled palynomorphs are predominantly Mississippian spores (up to 17% of the entire assemblage), with trace amounts of Permian–Triassic and Jurassic palynomorphs, indicating the age of strata that were being eroded in the Cordillera and supplying sediment to the foreland basin in the Upper Cretaceous. The trace Permian–Triassic and Jurassic palynomorphs help to constrain the likely source of the Permian detrital zircon grain because Triassic strata contain Permian-age detrital zircon (Fig 4). There is some uncertainty because Proterozoic rocks do not contain palynomorphs, and although proximate supersource provenance is possible the relative proportions of Precambrian age probabilities in Cretaceous samples do not significantly exceed those in the Mississippian spectrum. In summary, the proposition based on analysis of detrital zircon age probability patterns and geological context is therefore confirmed by a diagnostic proxy for the depositional age of the source strata.

## Conclusion

The supersource analysis traces the pathway of 2.0–1.8 Ga and 1.5–1.0 Ga detrital zircon from Proterozoic through Phanerozoic strata. Particularly in the case of 2.0–1.8 Ga grains that were at some point hosted by Paleoproterozoic sedimentary rocks, this shows that detrital zircons have very long, billion year scale, residence times in upper crustal sedimentary systems. Based on known occurrences of lower Paleozoic and Mesoproterozoic crystalline rocks a provenance interpretation for Upper Cretaceous samples might point to rocks in the Appalachian and Grenville orogens of eastern North America. A philosophical approach embracing sedimentary recycling, and the key methodology of cataloguing detrital zircon spectra from older strata *to identify potential sources*, clearly shows that all of the grains in question were most certainly recycled from older strata within the northern Cordillera. This methodology is confirmed by “detrital biostratigraphy” of recycled palynomorphs, and leads to a general proposition that sedimentary recycling plays a predominant role in detrital zircon provenance.

## Supporting Information

S1 Appendix Supplementary methods and results. (DOC)

S1 Fig Cretaceous U-Pb age-probability spectra. (PDF)

S2 Fig Neoproterozoic and Cambrian U-Pb age-probability spectra. (PDF)

S1 File Dataset of U-Pb detrital zircon age data. (XLSX)

S2 File Dataset of Correlation plots. (PDF)

S1 Table Data sources for manuscript figures. (PDF)

S2 Table Data sources for palynology. (PDF)
