# Supplementary material for: 1.8 Billion Years of Detrital Zircon Recycling Calibrates a Refractory Part of Earth’s Sedimentary Cycle
Source: PLoS One. 2015 Dec 14;10(12):e0144727. doi: 10.1371/journal.pone.0144727 (PMC4682852; doi:10.1371/journal.pone.0144727)
Supplement: S1 Appendix — (DOC) [file pone.0144727.s001.doc]

Appendix to Accompany “1.8 billion years of detrital zircon recycling calibrates a refractory part of Earth’s sedimentary cycle”

Hadlari, T. ^1^, Swindles, G.T. ^2^, Galloway, J.M. ^1^, Bell, K.M. ^3^, Sulphur, K.C. ^3^, Heaman, L.M. ^4^, Beranek, L.P. ^5^, Fallas, K.M. ^1^

1 *Geological Survey of Canada, 3303-33^rd^ St NW, Calgary AB, T2L 2A7*

[*thomas.hadlari@nrcan.gc.ca*](mailto:thomas.hadlari@nrcan.gc.ca) *(403) 292-7018*

[*jennifer.galloway@nrcan.gc.ca*](mailto:jennifer.galloway@nrcan.gc.ca)

[*karen.fallas@nrcan.gc.ca*](mailto:karen.fallas@nrcan.gc.ca)

2 *University of Leeds, Leeds, UK, LS2 9JT*

[*G.T.Swindles@leeds.ac.uk*](mailto:G.T.Swindles@leeds.ac.uk)

3 *University of Calgary, Calgary, Alberta* *T2N 1N4*

[*kmball@ucalgary.ca*](mailto:kmball@ucalgary.ca)

[*kyle.sulphur@NRCan.gc.ca*](mailto:kyle.sulphur@NRCan.gc.ca)

4 *University of Alberta, Edmonton, Alberta, T6G 2E3,*

[*larry.heaman@ualberta.ca*](mailto:Larry.Heaman@ualberta.ca)

5 *Memorial University of Newfoundland, St. John’s, Newfoundland, Canada*

[*lberanek@mun.ca*](mailto:lberanek@mun.ca)

**SUPPLEMENTARY METHODS AND RESULTS**

**U-Pb Detrital Zircon Geochronology: Methods**

Samples were pulverized using a jaw crusher and disk mill and a zircon concentrate obtained using standard mineral separation techniques (Wilfley Table, Frantz Isodynamic Separator, Heavy Liquids). In excess of 100 zircon grains were selected from each sample and secured in an epoxy mount and polished. An attempt was made to select zircon crystals that represented the range of crystal types present distinguished by variation in shape, colour and degree of rounding. Cathodoluminescence imaging of grains was not performed. The mounted zircon grains were analysed using a NewWave 213nm laser workstation coupled to a NuPlasma multi-collector inductively coupled plasma mass spectrometer (LA-MC-ICP-MS) at the University of Alberta. Analyses involved ablation of zircons using a 40 μm diameter laser spot size for 30 seconds. A “standard-sample-standard” method was used to correct instrumental drift during a single laser ablation session and involved analysis of an internal standard after every 12 unknown grains; this protocol was developed for provenance studies focusing on the dating of a large number of detrital zircon grains (Simonetti et al. 2005). Common Pb correction was applied using an initial Pb composition taken from Stacey and Kramers (1975). Complete data tables are listed S1 File Dataset 1.

Preferred ages are based on ^238^U/^206^Pb ages for zircon younger than 1000 Ma, and ^207^Pb/^206^Pb ages for older zircon. For interpretation we consider only ages that have concordance in the 95-105% interval (^238^U/^206^Pb age vs. ^207^Pb/^206^Pb age). Detrital zircon age results from this study (interpreted U-Pb ages and one standard deviation error) are presented in combined histogram and relative age probability plots using Microsoft Excel macros developed by G.E. Gehrels at University of Arizona (Fig. 3). Previously published detrital zircon U-Pb ages were compiled, subject to 95-105% concordance filter, and presented in relative age probability plots (Fig. 3). The relative probability plots use 1 standard deviation error whereas single ages are given as 2 standard deviations in the text.

**U-Pb Detrital Zircon Geochronology : Results**

***Little Bear Formation, Upper Cretaceous***

Samples of medium-grained sandstone were collected from the Little Bear Formation near the eastern front of the northern Cordillera (Fig. 1). Sample 08-TH-07D is from the lower part of the Little Bear Formation at the type section along Little Bear River (64.641 N, 126.325 W). Sample 10-HSB-514-B01 is from the upper portion of the Little Bear Formation at Little Bear River (64.785 N, 126.103 W).

***Results***

Analysis of sample 08-TH-07D yielded 33 of 109 grains, and sample 10-HSB-514-B01 yielded 28 of 110 grains, of detrital zircon that are considered for interpretation because the U-Pb ages are ≤ 5% discordant. Calculated ages from the two samples are very similar and so the sum of 61 analyses are treated as a combined sample of the Little Bear Formation detrital zircon population (Fig. DR1). Seven Archean ages range between 2970-2529 Ma and compose 11% of the combined sample. Three Paleoproterozoic probability highs have the ranges ca. 1986-1867 (7% of the sample), 1736-1713 Ma (8%), and 1654-1561 Ma (13%). A broad Mesoproterozoic age probability high consists of 20 ages between ca. 1494-1013 Ma composing 33% of the sample. A Neoproterozoic fraction is defined by 4 ages between ca. 941-909 Ma. A lower Paleozoic age grouping of ca. 466-412 Ma composes 10% of the sample and there is a single age of ca. 545 Ma. Two Early Carboniferous grains are ca. 355 Ma and 351. A single Permian grain is ca. 281 Ma. The youngest age cluster is defined by 3 grains of 92 ± 7 Ma, 91 ± 5 Ma, and 89 ± 5 Ma (2σ error).

The two samples described above have very similar detrital zircon ages as a sample of 40 grains from the upper portion of the Trevor Formation, which is considered a lateral stratigraphic equivalent to the lower Little Bear Formation (Hadlari et al., 2014). The Trevor Formation sample has age probability highs defined by grains that range ca. 2934-2672 Ma, 1968-1859 Ma, 1792-1716 Ma, 1320 Ma-994 Ma, 409-407 Ma, and a youngest grain of 94 ± 2 Ma. Published detrital zircon ages from the Trevor Formation are combined with results from the two samples presented here for a total of 101 grains in the age probability diagrams, statistical analysis, and subsequent discussion (Fig. 3).

**Statistical Analysis of detrital zircon data**

Aggregate similarity analysis such as the K-S test can be effective at comparing detrital zircon samples of the same depositional age because the range of possible ages is the same, that is, older than the age of deposition. The aggregate similarity approach breaks down when comparing samples of different depositional age because the younger sample is likely to contain age fractions that are impossible in the older sample. The result of comparing samples of different age can be low aggregate similarity even if the older age fractions are identical, which does little to address the possibility of sediment recycling and inheritance of fractions within the overall age spectrum.

The various Proterozoic and Cambrian detrital zircon samples considered here are subdivided into two “supersource” groups based on the predominance of Paleoproterozoic 2000-1800 Ma or Mesoproterozoic 1500-1000 Ma ages (see main text). Figure DR2 shows the U-Pb age spectra of Supersource I comprising the Snowcap Assemblage of Yukon Tanana Terrane (Piercey and Colpron, 2009), the Wernecke Supergroup (Furlanetto et al., 2009), and Cambrian derivatives (Illtyd and Slats formations, Lane and Gehrels, 2014). Supersource II comprises the Mackenzie Mountains Supergroup (Rainbird et al., 1997; Villeneuve et al., 1998) and its Cambrian derivatives (Hadlari et al., 2012) (Fig. DR2). The Phanerozoic samples are grouped based on signature and published tectono-stratigraphic affinity (see Table 1, main text). All sample groups have n numbers that exceed the recommended minimum of n=95-100 for statistical treatment (Vermeesch, 2004; Andersen, 2005), except the Devonian West grouping (n=61), which only has the requisite number for a general age-probability characterization of the main zircon fractions (Vermeesch, 2004).

***Running (time window) correlation analysis: methods***

Bivariate running correlation analysis (Pearson r) was used to determine the correlation between age probability distributions, and the temporal variation of the correlation. Relative U-Pb age probability distributions were standardised to z-scores prior to analysis. A time window of 100 Ma was used. A *p* = 0.05 significance level for each running correlation was calculated using a series of Monte Carlo simulations to determine the null distribution (as standard significance testing is arguably inappropriate due to the multiple comparison problem). A running correlation was calculated for many different random variables and the highest correlation for each trial was determined. This approach yields more suitable critical values for running correlations – albeit slightly higher (harder to pass) than critical values for standard Pearson correlation tests. As well as the Monte Carlo approach the statistical significance of the correlation was calculated using a classical approach - the t-distribution with n-2 degrees of freedom (critical value R = 0.1, p = 0.05, n = 400). This was considerably lower than the Monte Carlo approach (R = ~0.3) in all cases. However, in reality, there was little difference between overall the ‘bands’ deemed significant or non-significant when we compared the classical and Monte Carlo-based critical values. The analysis was carried out using the gtools library (Warnes et al., 2014) in R (R Core Team, 2014). An example of the code for this analysis (4000 data points and window size of 100) is as follows:

#monte-carlo test

res<-replicate(10000, {

y<-rnorm(4000)

z<-max(running(x,y, fun=cor, width=100))# find maximum correlation

})

quantile(res, 0.95) #critical of maximum correlation at p=0.05.

The running time window correlation method is well suited to identifying combinations of detrital zircon sources because it correlates trends within sliding time windows of relative U-Pb age probability. The results show the time (Ma) intervals that have correlative age probability trends between sets of two samples. The relative U-Pb age relative probabilities were chosen for this analysis because they contain all ages and 1σ errors for each sample set. Care should be taken because trends are correlated irrespective of magnitude and so the user has to determine how important correlations are for low relative age probabilities. In this contribution, space under probability curves is colour coded in order to scale the graphical presentation of the correlation interval to the relative probability thereby showing the low or even negligible importance of some low probability intervals. A second point addresses comparison of precise vs imprecise data. Precise age data will be “spiky” whereas less precise data will be more smooth and the result will be a discontinuous correlation line bar, and for this reason the user should not consider relatively small breaks in the correlation to be a negative result. Most sample groupings exceed recommended n numbers for statistical treatment (n > 95), except for the Devonian West group. At n=61, the overall shape of the probability curve should be characterized but some low probability age fractions may have been missed (those composing less than 8.5% of the population), and so statistical correlations for the Devonian West group are considered to be generalizations.

***Running (time window) correlation analysis: results***

Each of the supersource U-Pb age probability spectra and the Cretaceous spectrum were tested by running time window analysis for correlation to each of the age probability spectra of the Phanerozoic sample sets. The resulting twenty two correlation plots are included as S2 File Dataset 2. Time intervals of statistically significant correlation were converted to a line bar in Ma as in Step 1 of Fig. 3. The line bar was then used to colour code intervals of U-Pb age probability in the Phanerozoic U-Pb age spectra, shown as Step 2 in Fig. 3. As discussed in the main text, the age probability intervals that are most relevant for correlation to supersource and U. Cretaceous age spectra are shown in Fig. 4 of the main manuscript.

**Palynology: Methods**

Palynomorphs have degradation resistant walls composed of organic molecules (sporopollenin) making them excellent candidates for fossilization and recycling (Traverse, 1994). Their ability to survive cycles of recycling and redeposition can provide information about sediment provenance (Traverse, 2007). Preserved palynomorphs were studied from the Little Bear Formation of the Husky et al. Sah Cho L-71 (300/L-71-6430-12530/0 [NT]) exploration well from the Mackenzie Valley, NWT (Latitude 64º 20' 41.70" N; Longitude 125º 44' 40.54" W; UTM Zone 10W; Easting 367444.8 m; Northing 7138312.6 m; NAD83). Twenty samples of fine grained clastic (primarily mudstone and siltstone) washed cuttings were collected in 25 m intervals from this exploration well with permission from the National Energy Board. One outcrop sample of the Turonian portion of the Trevor Formation, 07-TH-25N, collected in 2007 is also included (65.419 N, 130.010 W).

Samples were prepared at Geological Survey of Canada, Calgary (GSC) following to standard extraction techniques (Wood et al. 1996), including acid digestion, heavy liquid separation, oxidation, and staining. Palynomorph taxa were identified and counted using an Olympus Vanox-T transmitted light microscope at 1000x magnification and an Olympus BX61 transmitted light microscope at 400x and 1000x magnification. Identification of recycled palynomorphs was achieved through knowledge of taxonomic ranges and augmented where possible by differences in thermal alteration between recycled and in situ palynomorphs (Pearson, 1984). A minimum of 100 palynomorphs were counted for each unsieved sample. Residues and slides are contained in the permanent collections of the Geological Survey of Canada.

**Palynology: Results**

The Little Bear Formation of the Mackenzie Valley contains both in situ Upper Cretaceous palynomorphs and populations of recycled Paleozoic and Mesozoic palynomorphs (Bell, 2014, 2015). Carboniferous palynomorphs are most commonly recycled, however, rare recycled Jurassic and Early-mid Cretaceous dinoflagellates and recycled Permian-Early Triassic taeniate bisaccate pollen are also present in some of the samples (Fig. 5.4). Recycled palynomorphs from the sampled interval of the Little Bear Formation comprises approximately 4%-23% of the total palynomorph assemblage. In particular, diagnostic Carboniferous spore taxa were found in all samples analyzed from the Little Bear Formation, and compose approximately 1% to 17% of the entire assemblage. The Carboniferous assemblage found the Little Bear Formation includes spore genera such as *Densosporites*, *Tumulispora*, *Diatomozonotriletes*, *Waltzispora*, and *Triquitrites* (Bell, 2014).

Similarly, the Trevor Formation also contains Carboniferous spores, commonly including *Densosporites* and rarely *Triquitrites* (Sulphur, 2014). Recycled palynomorphs compose approximately 20% of the Trevor Formation assemblage. The presence of spore types with strong equatorial features such as *Densosporites*, *Diatomozonotriletes*, *Triquitrites* and *Waltzispora* in both the Little Bear and Trevor formations suggests suggest that both assemblages are most likely of Lower Carboniferous (Mississippian) age.

**REFERENCES CITED**

Andersen, T., 2005. Detrital zircons as tracers of sedimentary provenance: limiting conditions from statistics and numerical simulation. Chemical Geology, 216, p. 249-270.

Beikman, H.M., (compiler) 1980, Geologic map of Alaska: U.S. Geological Survey, scale 1:2 500 000, 1 sheet.

Bell, K.M., 2014. Applied research report on 30 cutting samples from Sah-Cho L-71 exploration well, Mackenzie Corridor, Northwest Territories (NTS 096C/05). Geological Survey of Canada, Paleontological Report KMB-2014-01, Tables 1-3, 29 p.

Bell, K.M. 2015. Applied research report on 20 cutting samples from the Little Bear Formation of Sah Cho L-71 exploration well, Mackenzie Corridor, Northwest Territories (NTS 096C/05). Geological Survey of Canada, Paleontological Report KMB-2015-01, 20 p.

Beranek, L.P. and Mortensen, J.K., 2011, The timing and provenance of the Late Permian Klondike orogeny in northwestern Canada and arc-continent collision along western North America: Tectonics, v. 30, TC5017, 23 p.

Beranek, L.P., Mortensen, J.K., Lane, L.S., Allen, T.L., Fraser, T.A., Hadlari, T., and Zantvoort, W.G. 2010a, Detrital zircon geochronology of the western Ellesmerian clastic wedge, northwestern Canada: Insights on Arctic tectonics and the evolution of the northern Cordillera miogeocline: Geological Society of America Bulletin, v. 122, p. 1899-1911.

Beranek, L.P., Mortensen, J.K., Orchard, M.J., and Ullrich, T., 2010b, Provenance of North American Triassic strata from west-central and southeastern Yukon: correlations with coeval strata in the Western Canada Sedimentary Basin and Canadian Arctic Islands: Canadian Journal of Earth Sciences, v. 47, p. 53-73.

Colpron, M., Nelson, J.L. and Murphy, D.C., 2006, A tectonostratigraphic framework for the pericratonic terranes of the northern Cordillera, In Colpron, M. and Nelson, J.L., eds., Paleozoic Evolution and Metallogeny of Pericratonic Terranes at the Ancient Pacific Margin of North America, Canadian and Alaskan Cordillera: Geological Association of Canada, Special Paper 45, p. 1-23.

Furlanetto, F., Thorkelson, D.J., Davis, W.J., Hibson, H.D., Rainbird, R.H., and Marshall, D.D., 2009, Preliminary results of detrital zircon geochronology, Wernecke Supergroup, Yukon, in Weston, L.H., Blackburn, L.R., and Lewis, L.L., eds., Yukon Exploration and Geology 2008: Whitehorse, Canada, Yukon Geological Survey, p. 125–135.

Hadlari, T., Davis, W.G., Dewing, K., Heaman, L.M., Lemieux, Y., Ootes, L., Pratt, B.R., and Pyle, L.J., 2012, Two detrital zircon signatures for the Cambrian passive margin of Laurentia highlighted by new U-Pb results from Northwest Territories, Canada: Geological Society of America Bulletin, v. 124, p. 1155-1168.

Hadlari, T., MacLean, B., Galloway, J.M., Sweet, A.R., White, J.M., Thomson, D., Schröder-Adams, C., and Gabites, J., 2014, The flexural margin, the foredeep, and the orogenic margin of a northern Cordilleran foreland basin: Cretaceous tectonostratigraphy and detrital zircon provenance, northwestern Canada: Marine and Petroleum Geology, v. 58, p. 173-186.

Jakobsson, M., Macnab, R., Mayer, L., Anderson, R., Edwards, M., Hatzky, J., Schenke, H.W., and Johnson, P., (2008). An improved bathymetric portrayal of the Arctic Ocean: Implications for ocean modeling and geological, geophysical and oceanographic analyses. Geophysical Research Letters, 35, 5 p. doi:10.1029/2008GL033520

Lane, L.S. and Gehrels, G.E., 2014, Detrital zircon lineages of late Neoproterozoic and Cambrian strata, NW Laurentia: Geological Society of America Bulletin, v. 126, p. 398-414.

Lemieux, Y., Hadlari, T., and Simonetti, A., 2011, Detrital zircon geochronology and provenance of Devono-Mississippian strata in the northern Canadian Cordilleran miogeocline: Canadian Journal of Earth Sciences, v. 48, p. 515-541.

Pearson, D.L. 1984, Pollen/spore color ‘standard’, version 2: Phillips Petroleum Company, Bartlesville, OK, Exploration Projects Section.

Piercey, S.J. and Colpron, M., 2009, Composition and provenance of the Snowcap assemblage, basement to the Yukon-Tanana terrane, northern Cordillera: Implications for Cordilleran crustal growth: Geosphere, v. 5, p. 439-464.

Plafker, G., Gilpin, L.M., and Lahr, J.C., 1994, Neotectonic map of Alaska, in Plafker, George, and Berg, H.C., eds., The Geology of Alaska: Geological Society of America, 2 sheets, scale 1:2,500,000.

R Core Team 2014, R: A language and environment for statistical computing. R Foundation for Statistical Computing, Vienna, Austria. URL http://www.R-project.org/.

Rainbird, R.H., Heaman, L.M., and Young, G., 1992, Sampling Laurentia: detrital zircon geochronology offers evidence for an extensive Neoproterozoic river system originating from the Grenville orogen: Geology, vol. 20, p. 351-354.

Rainbird, R.H., McNicholl, V.J., Theriault, R.J., Heaman, L.M., Abbott, J.G., Long, D.G.F., and Thorkelson, D.J., 1997, Pan-continental river system draining Grenville Orogen recorded by U-Pb and Sm-Nd geochronology of Neoproterozoic quartzarenites and mudrocks, northwestern Canada: The Journal of Geology, v. 105, p. 1-17.

Simonetti, A., Heaman, L.M., Hartlaub, R.P., Creaser, R.A., McHattie, T., and Böhm, C., 2005, Rapid and precise U-Pb dating by laser ablation MC-ICP-MS using new multiple ion counting-faraday collector array: Journal of Analytical Atomic Spectroscopy, vol. 20, p. 677-686.

Stacey, J.S. and Kramers, J.D., 1975, Approximation of terrestrial lead isotope evolution by a two-stage model: Earth and Planetary Science Letters, vol. 26, p. 207-221.

Sulphur, K.C., 2014. Palynological report on 27 samples submitted by Thomas Hadlari, Geological Survey of Canada, Calgary, collected from Martin House, Arctic Red, Slater River, and Trevor formations (Albian to Cenomanian), Hume River, Northwest Territories (NTS 106H/5). Geological Survey of Canada, Paleontological Report KCS-2014-01, 6 p.

Traverse, A. 1994, Sedimentation of Organic Particles. Cambridge University Press: 1-544.

Traverse, A. 2007, Paleopalynology, 2nd Edition. In: Topics in Geobiology 28. Springer: 1-813.

Vermeesch, P., 2004, How many grains are needed for a provenance study?: Earth and Planetary Science Letters, vol. 224, p. 441-451.

Villeneuve, M.E., Butterfield, N.J., Cook, D.G., MacLean, B.C. and Rainbird, R.H., 1998, Age of the Cap Mountain (Northwest Territories) Proterozoic section, sequence B, based on detrital zircon ages: Radiogenic Age and Isotopic Studies Report 11, Geological Survey of Canada, Current Research 1998-F, p. 117-127.

Warnes, G.R., Bolker, B. and Lumley, T. (2014). gtools: Various R programming tools. R package version 3.4.1. http://CRAN.R-project.org/package=gtools

Wheeler, J.O., Hoffman, P.F., Card, K.D., Davidson, A., Sanford, B.V., Okulitch, A.V., and Roest, W.R., (compilers) 1996, Geological Map of Canada, Geological Survey of Canada, Map D1860A, scale 1:5 000 000.

Wood, G.D., Gabriel A.M. and Lawson, J.C. 1996, Palynological techniques – Significance of maceration and microscopical innovations to integrated studies: Amoco Production Company, p. 1-81.

SUPPLEMENTARY FIGURE CAPTIONS

**Figure DR1:** Relative probability plot for detrital zircon ages from two Cretaceous samples reported here.

**Figure DR2:** Relative probability plots for detrital zircon ages for Proterozoic and Cambrian samples of the northern Cordillera.

SUPPLEMENTARY TABLE CAPTIONS

**Table DR1:** List of references for data sources in figures 1-2.

**Table DR2:** List of palynology sample information for Figure 4.
