# Supplementary figures and images for: 1.8 Billion Years of Detrital Zircon Recycling Calibrates a Refractory Part of Earth’s Sedimentary Cycle
Source: PLoS One. 2015 Dec 14;10(12):e0144727. doi: 10.1371/journal.pone.0144727 (PMC4682852; doi:10.1371/journal.pone.0144727)

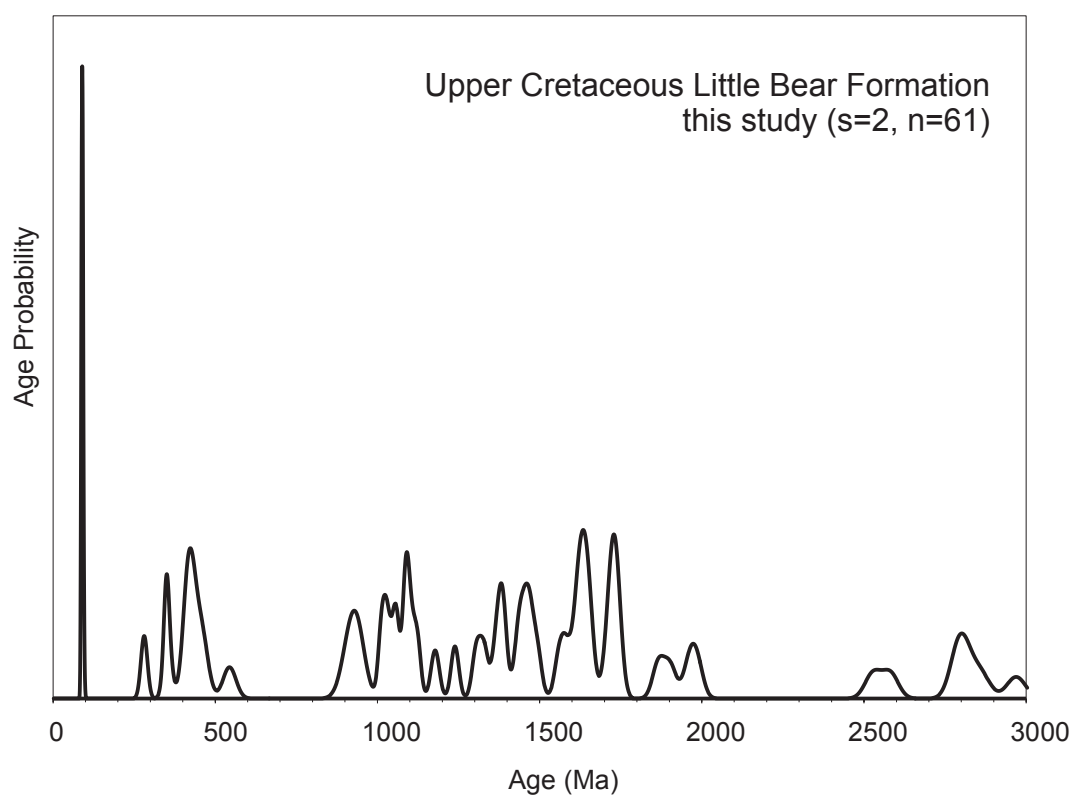

S1 Figure 1

Supplement: S1 Fig — (PDF) [file pone.0144727.s002.pdf]

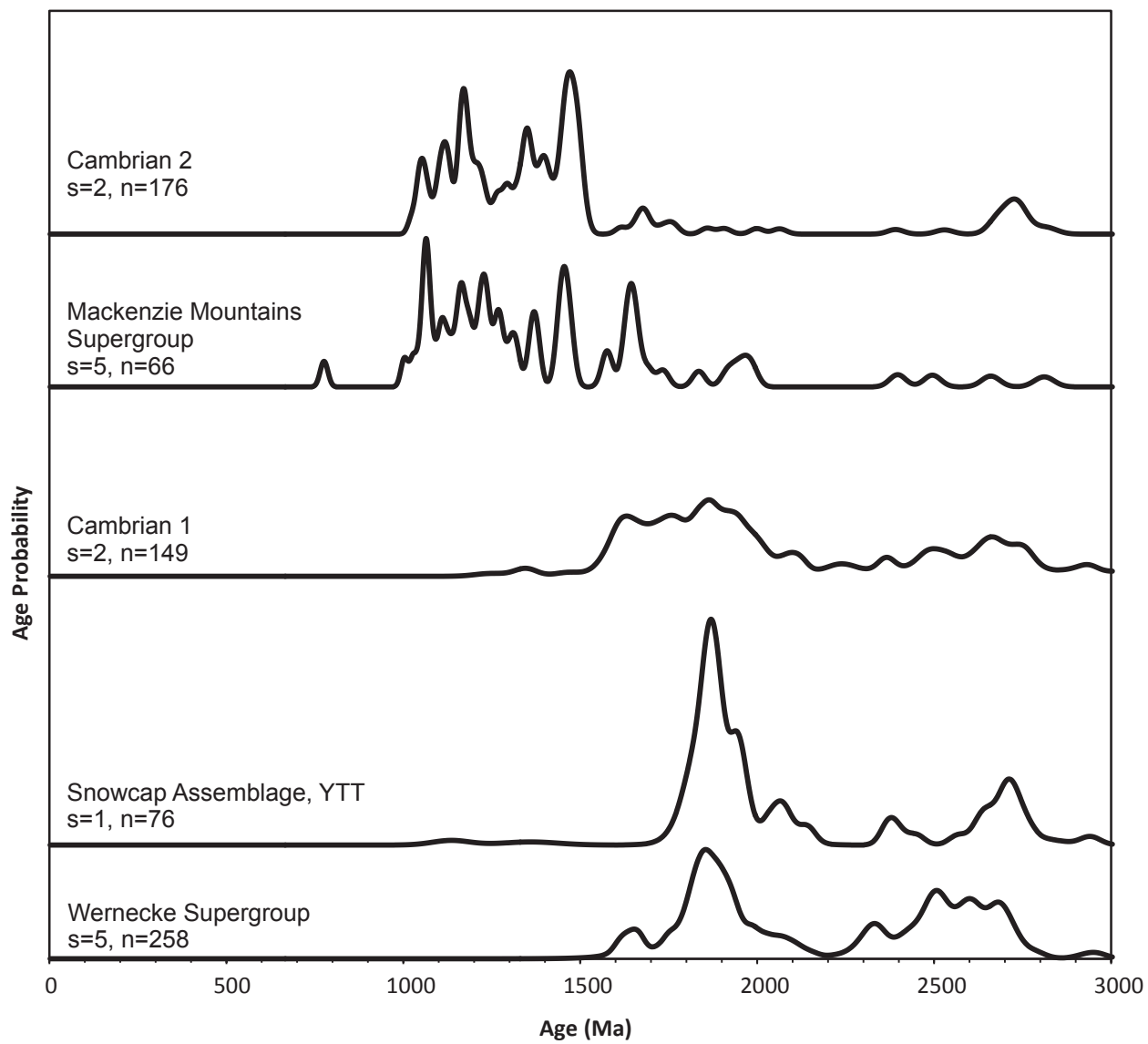

S2 Figure 2

Supplement: S2 Fig — (PDF) [file pone.0144727.s003.pdf]
