# Supplementary material for: 1.8 Billion Years of Detrital Zircon Recycling Calibrates a Refractory Part of Earth’s Sedimentary Cycle
Source: PLoS One. 2015 Dec 14;10(12):e0144727. doi: 10.1371/journal.pone.0144727 (PMC4682852; doi:10.1371/journal.pone.0144727)
Supplement: S2 File — (PDF) [file pone.0144727.s005.pdf]

From Figure 3, the labels in the correlation plots correspond to:

SOU1 = Supersource I
SOU2 = Supersource II
DEVW = Devonian West
DEVE = Devonian East
LMT = Lower Mississippian
UMK = Upper Mississippian
TRIF = Triassic Interior Platform
TYO = Triassic Yukon-Tanana terrane overlap assemblage
UCRET = Upper Cretaceous

The colour of the red and black curves correspond to the colour of the labels.

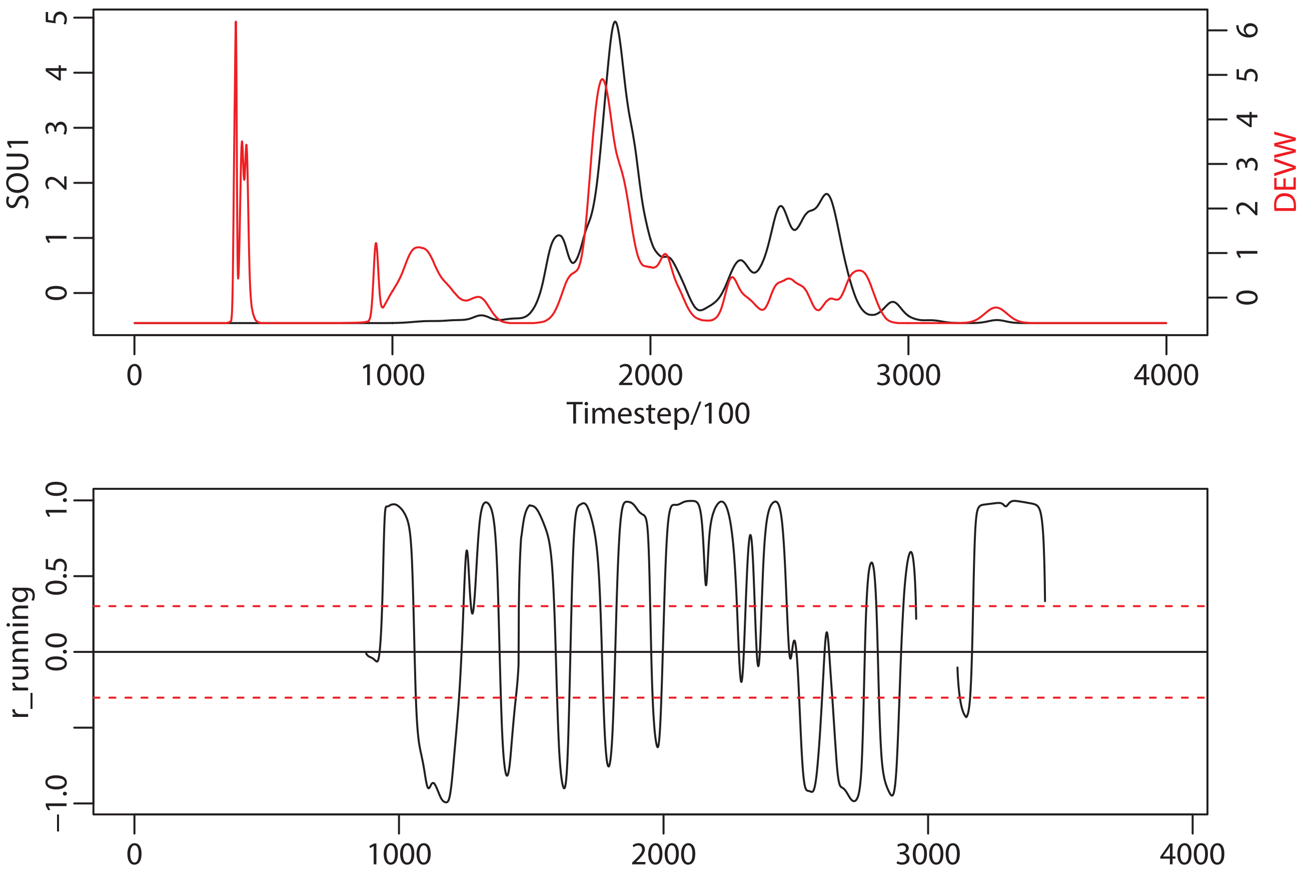

SOU1
DEVW
Timestep/100
r_running
0
1000
2000
3000
4000

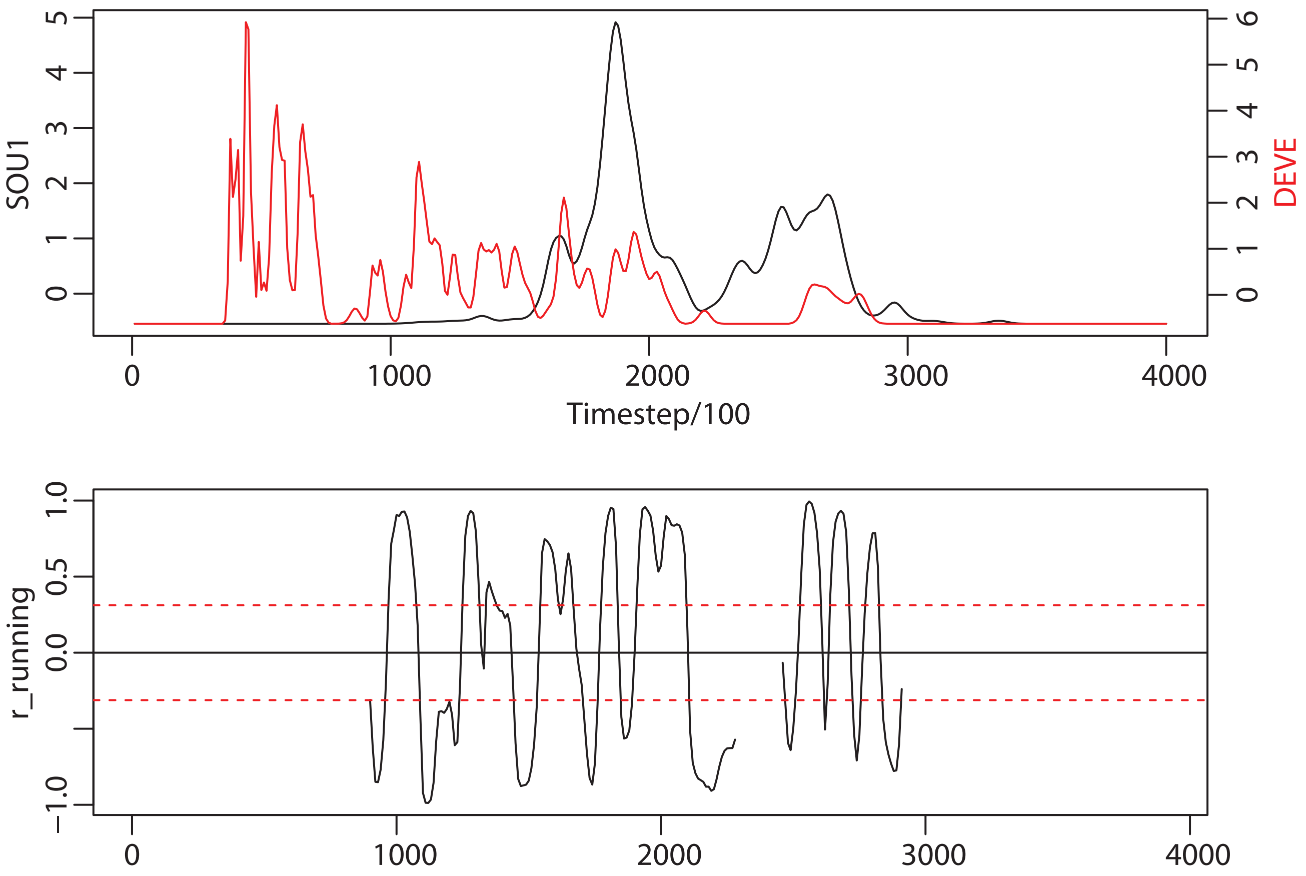
SOU1
DEVE
Timestep/100
r_running

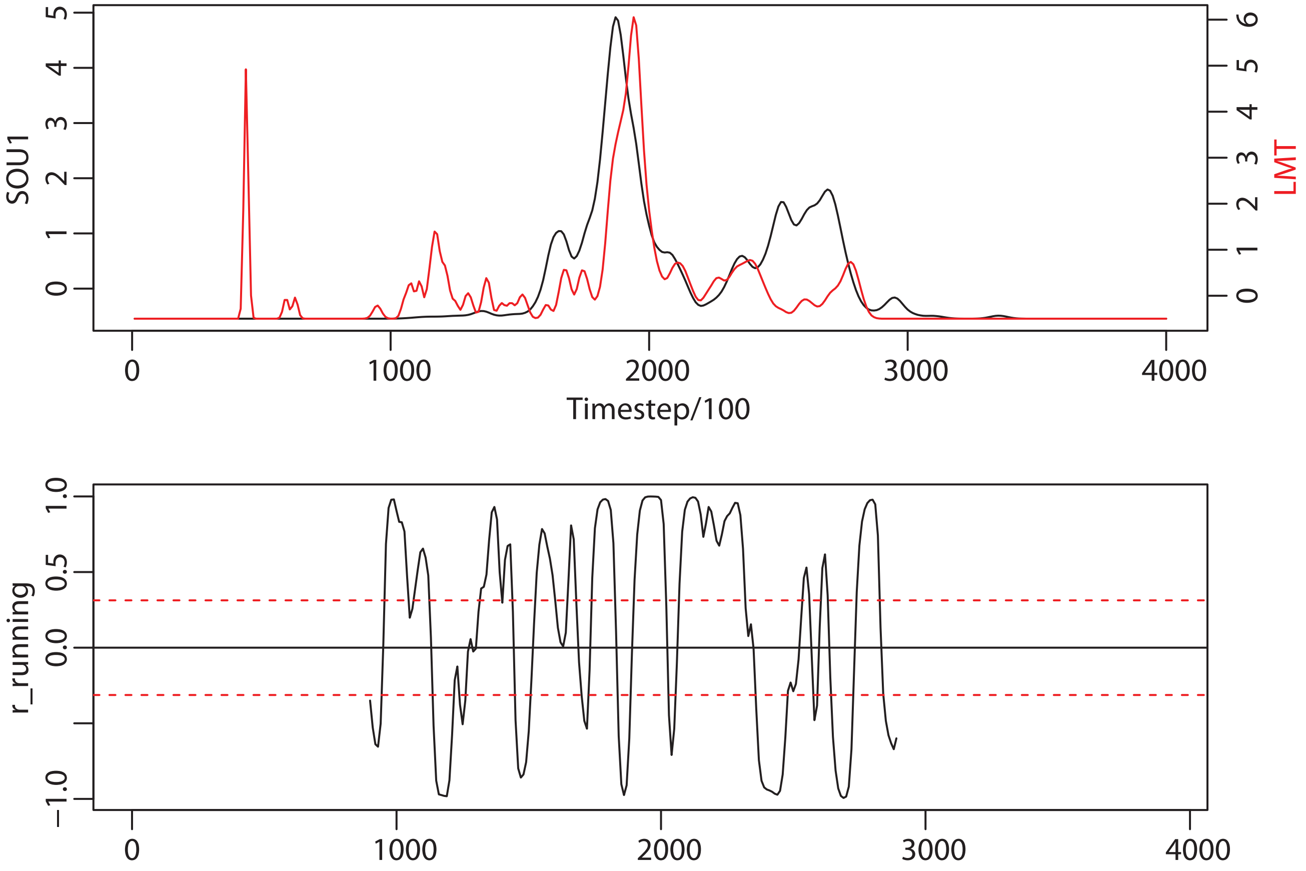

SOU1
LMT
Timestep/100
0
1000
2000
3000
4000
r_running
1.0
0.5
0.0
−1.0

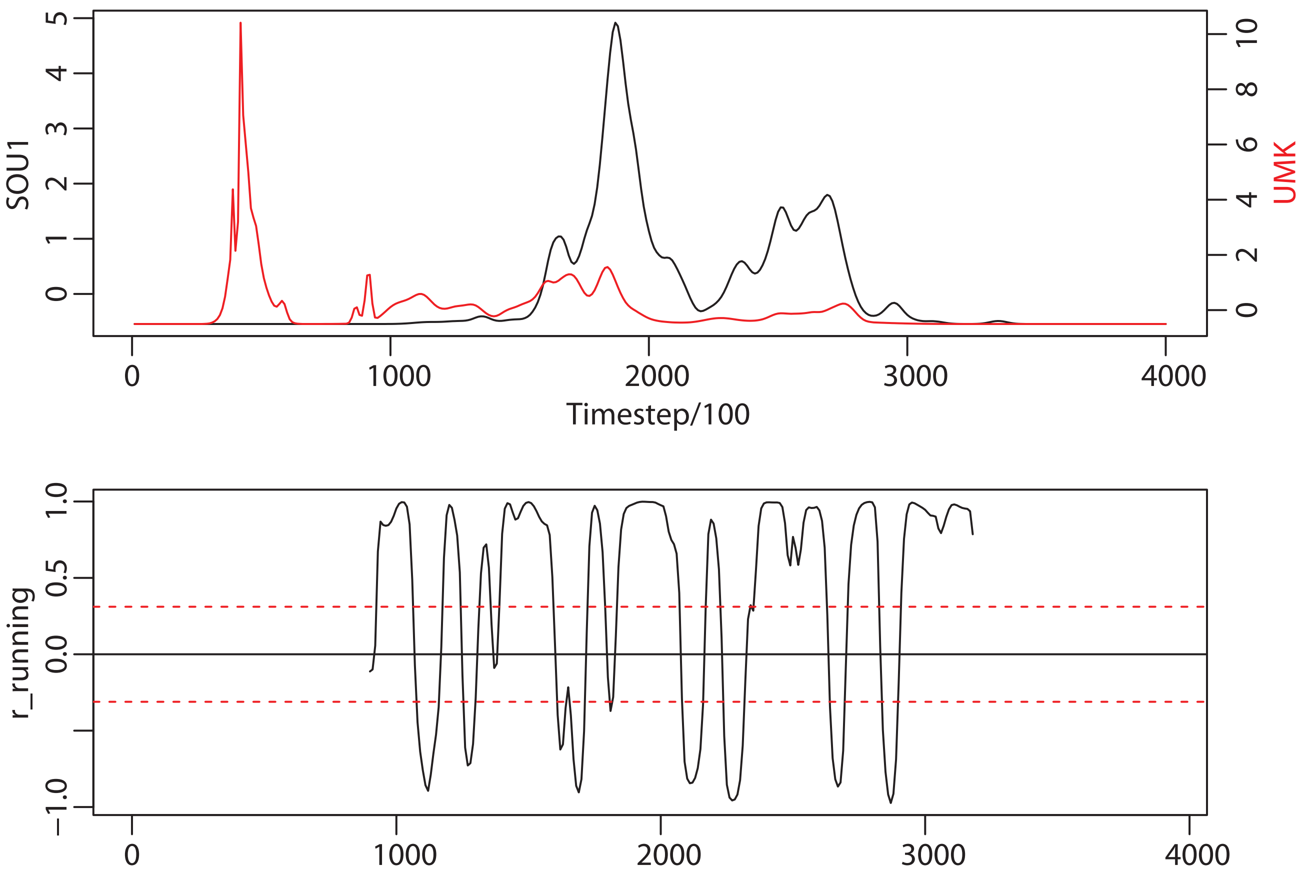
SOU1
UMK
Timestep/100
r_running

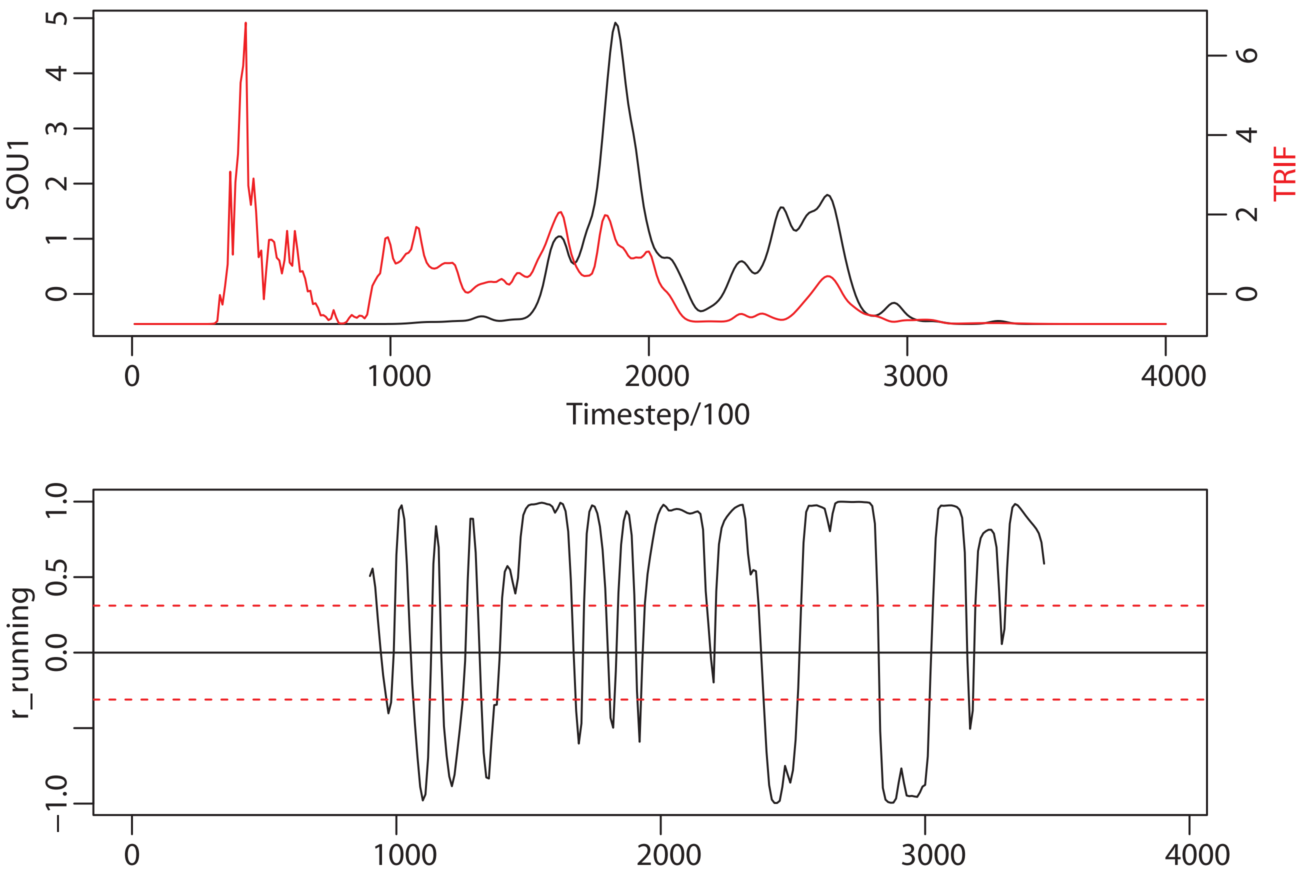

SOU1
TRIF
Timestep/100
r_running

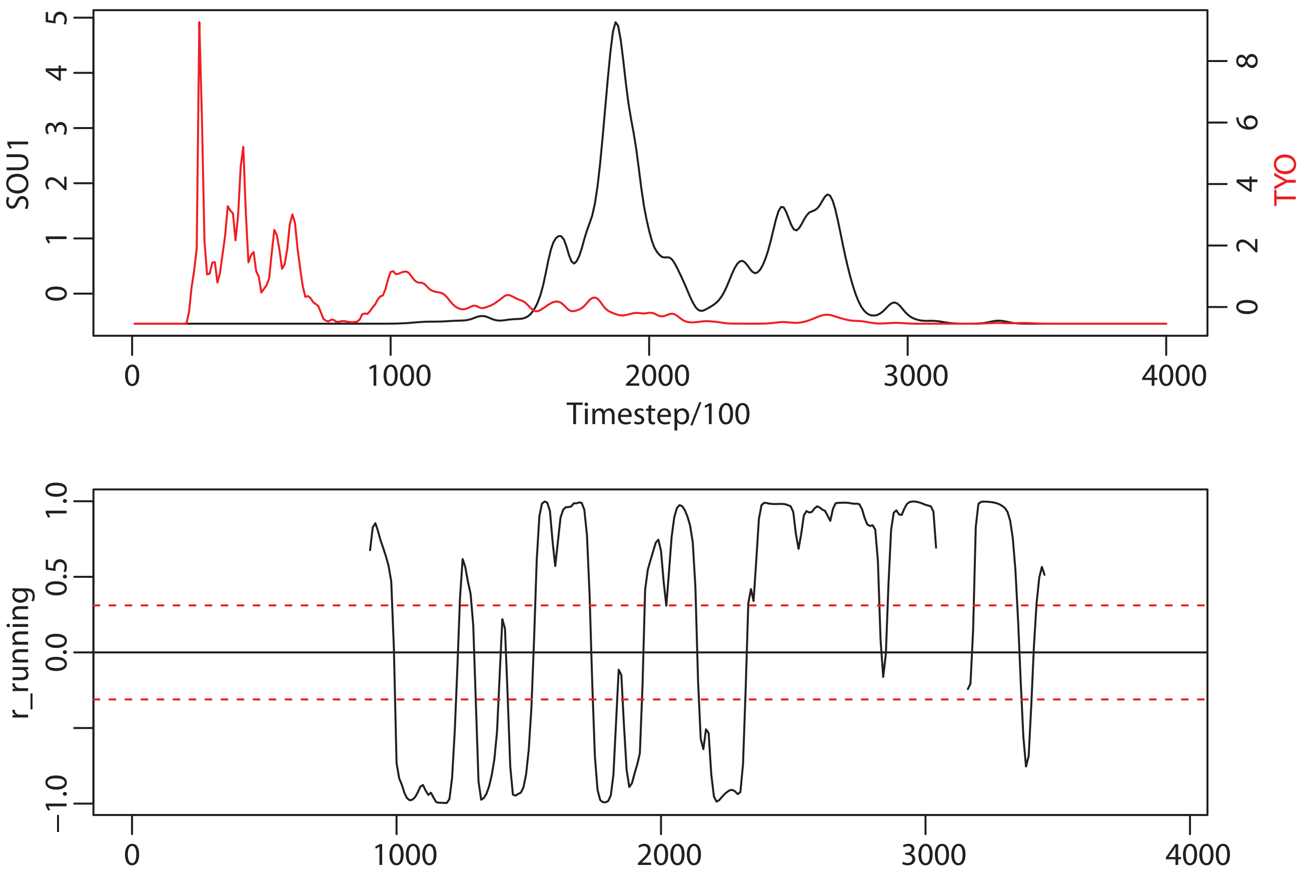
SOU1
TYO
Timestep/100
r_running
0
1000
2000
3000
4000
0
1
2
3
4
5
0
2
4
6
8
−1.0
0.0
0.5
1.0

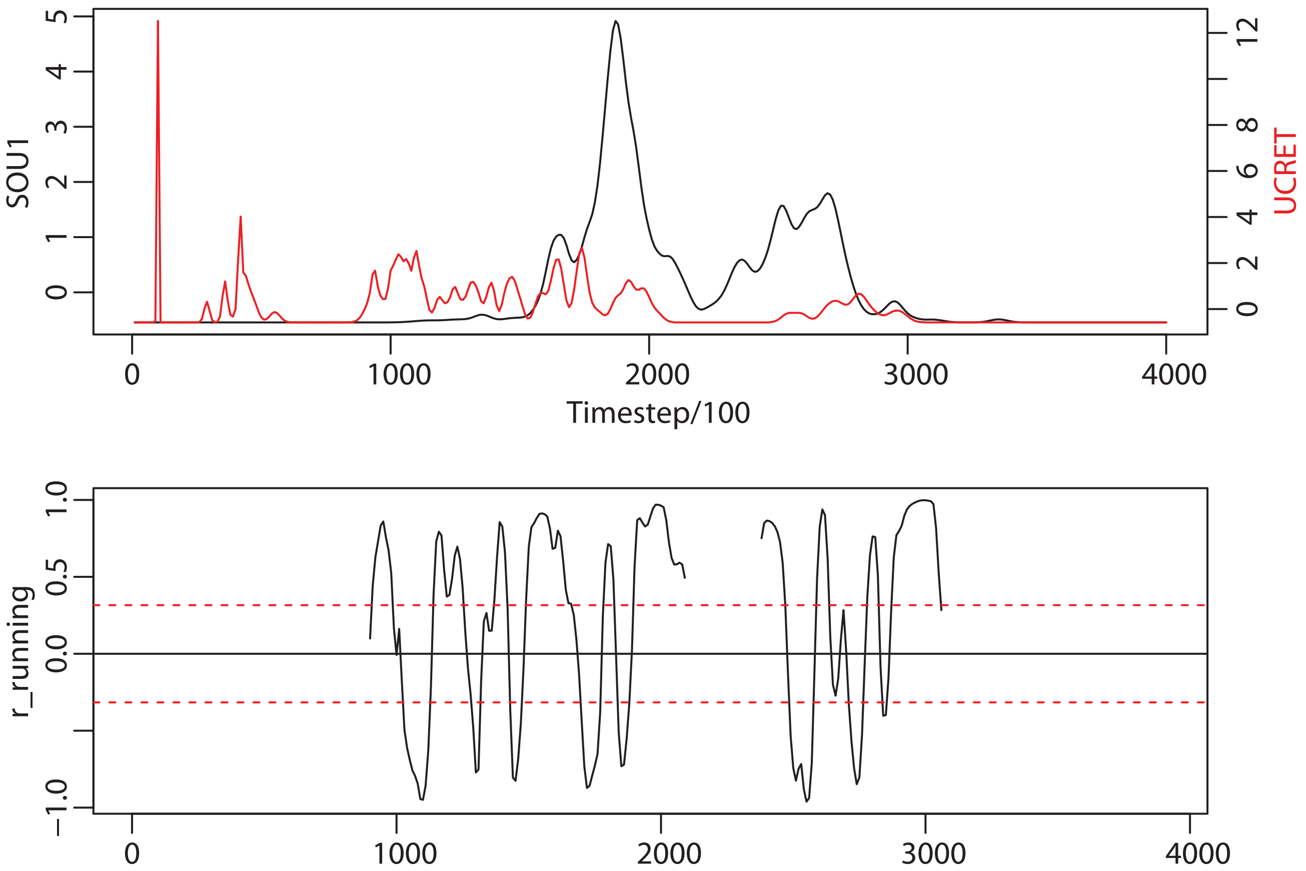

SOU1
UCRET
Timestep/100
r_running

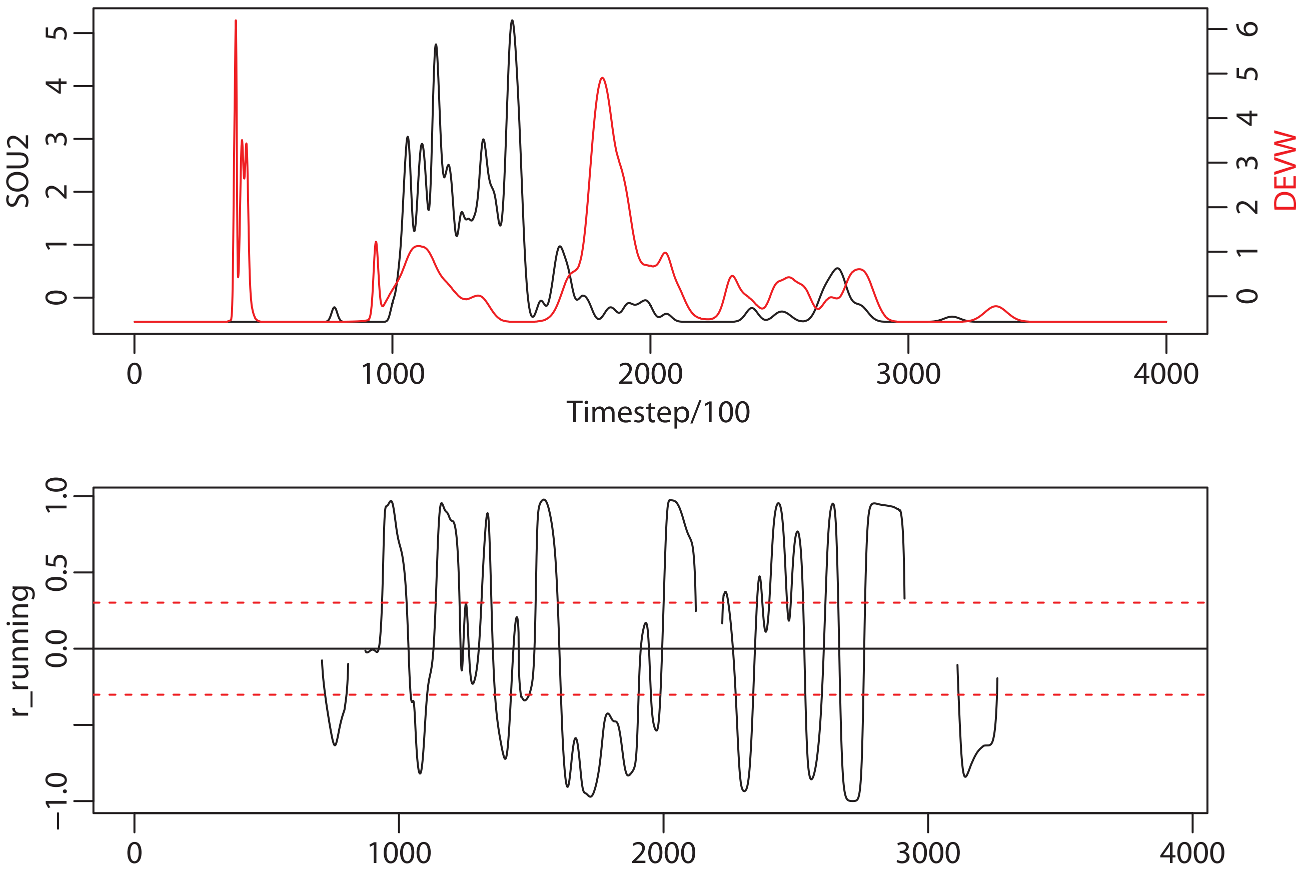
SOU2
DEWW
Timestep/100
r_running
0
1000
2000
3000
4000

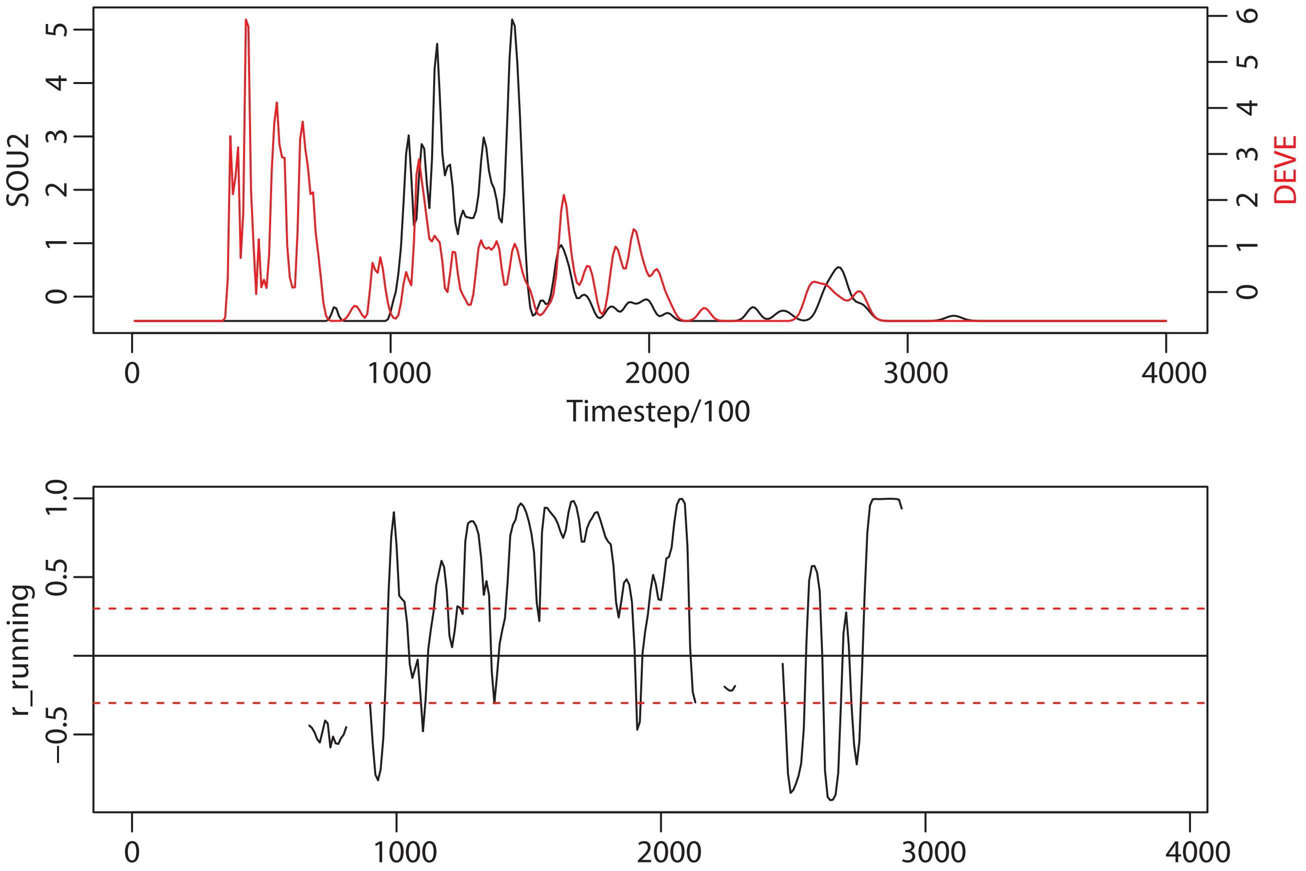

SOU2
DEVE
Timestep/100
r_running
0
1000
2000
3000
4000

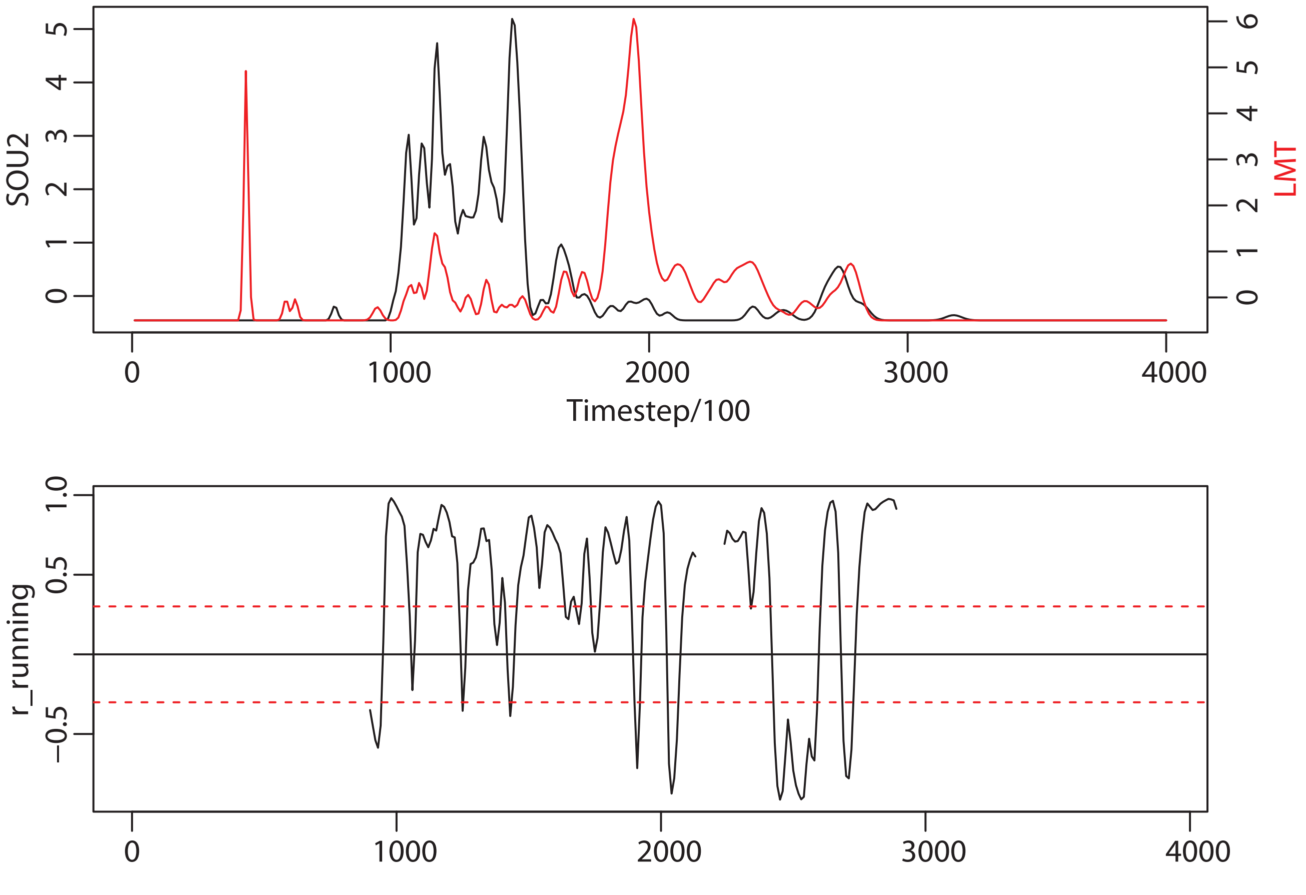

SOU2
LMT
Timestep/100
0 1000 2000 3000 4000
0 1 2 3 4 5
0 1 2 3 4 5 6
r_running
−0.5 0.5 1.0
0 1000 2000 3000 4000

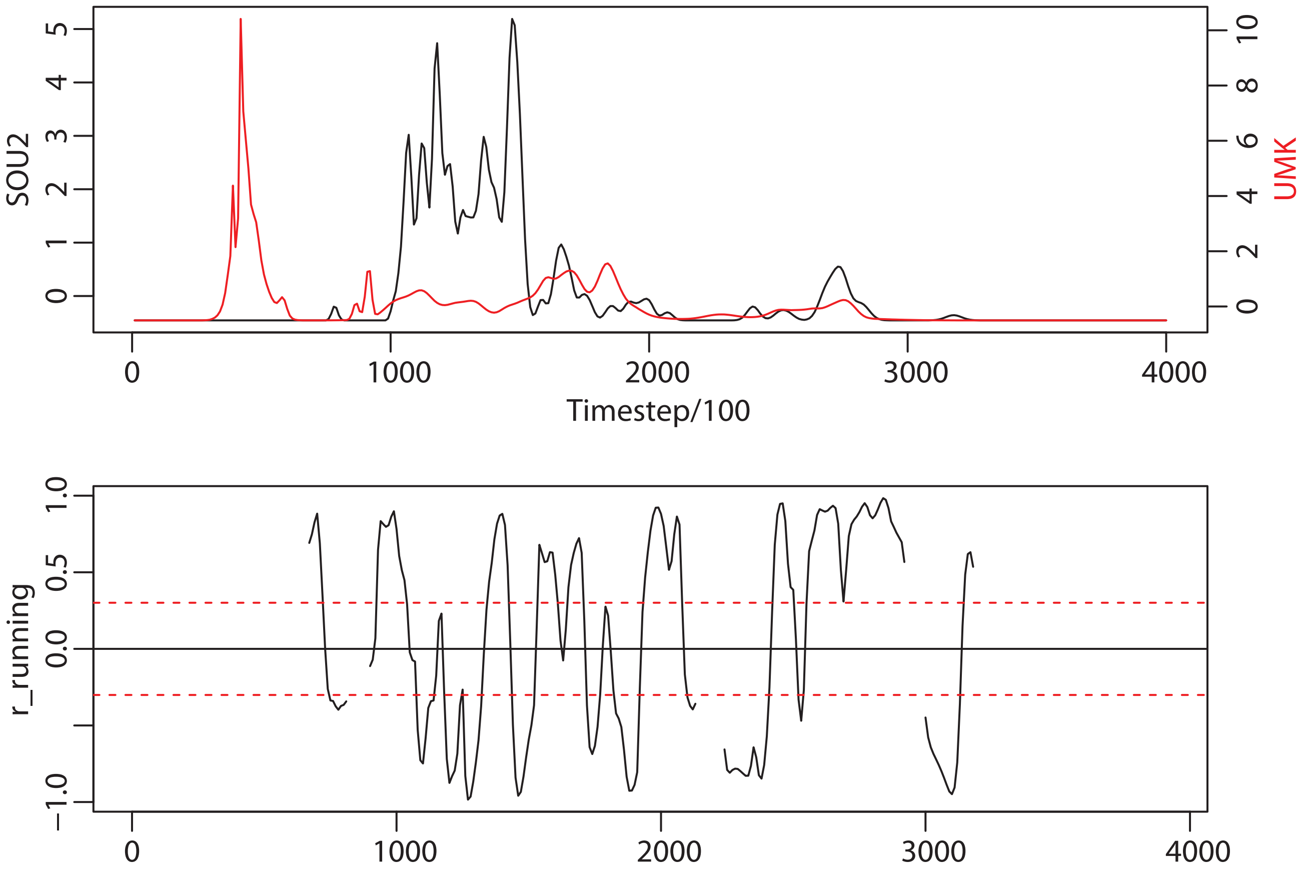

SOU2
UMK
Timestep/100
0
1000
2000
3000
4000
r_running
1.0
0.5
0.0
−1.0

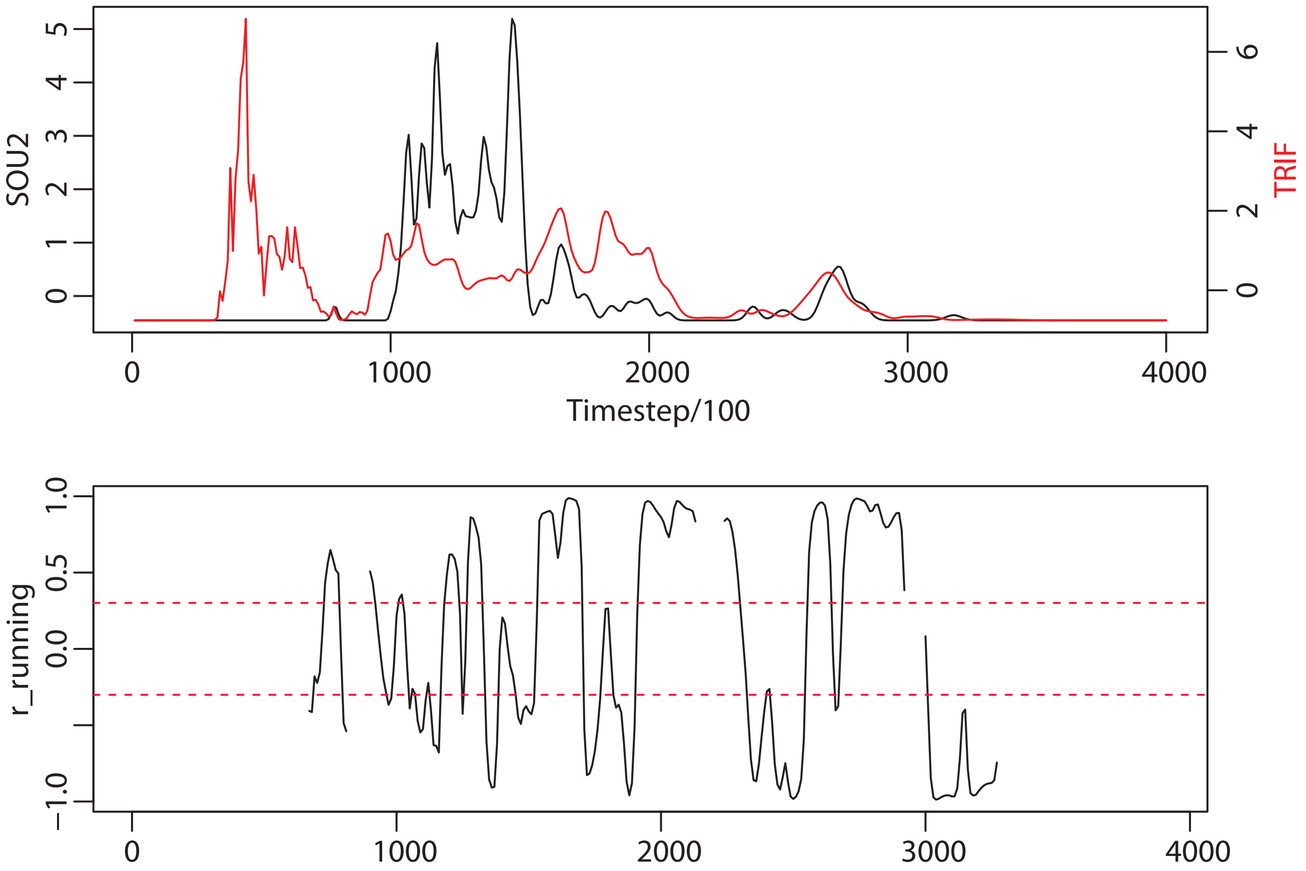

SOU2
TRIF
Timestep/100
r_running

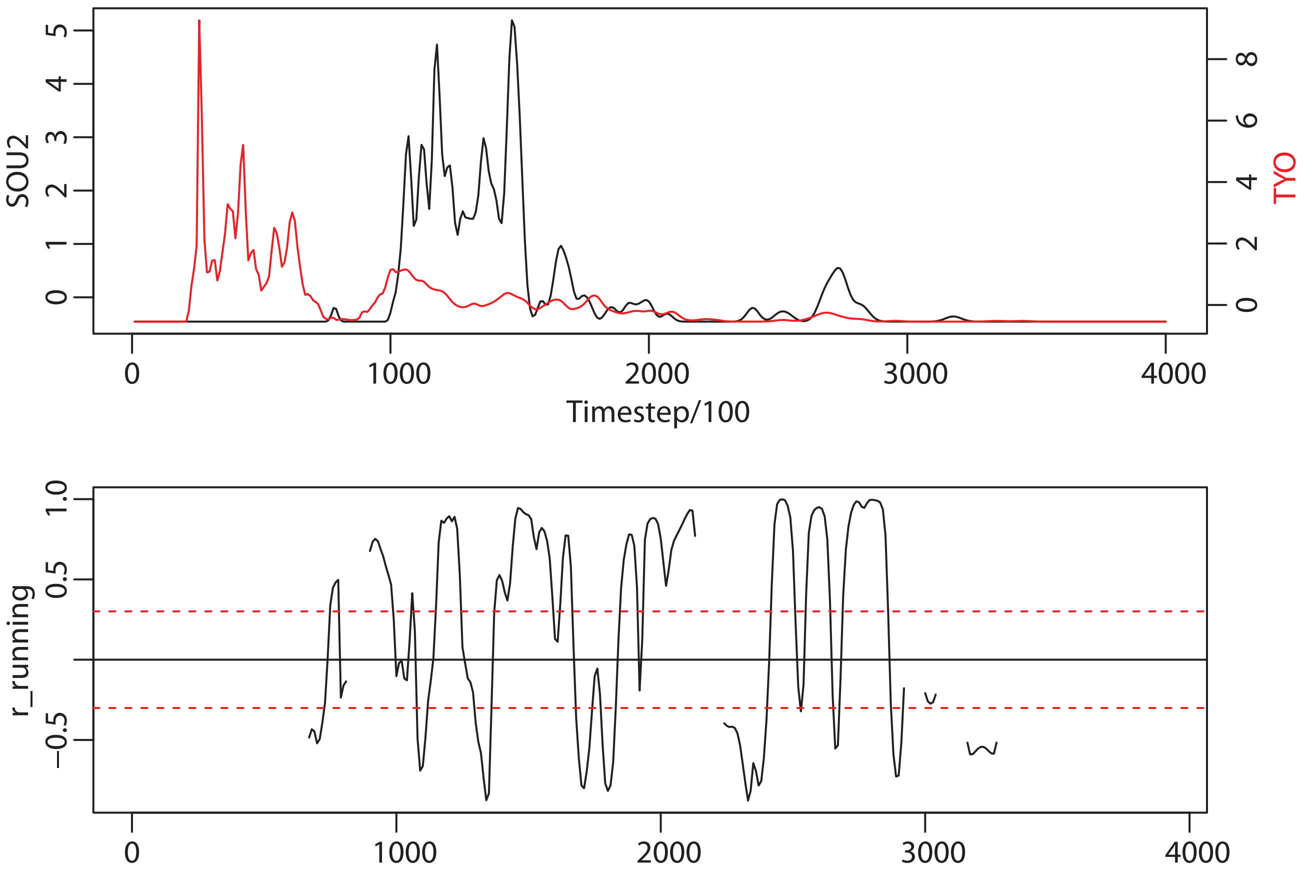

SOU2
TYO
Timestep/100
r_running
0
1000
2000
3000
4000
5
4
3
2
1
8
6
1.0
0.5
−0.5

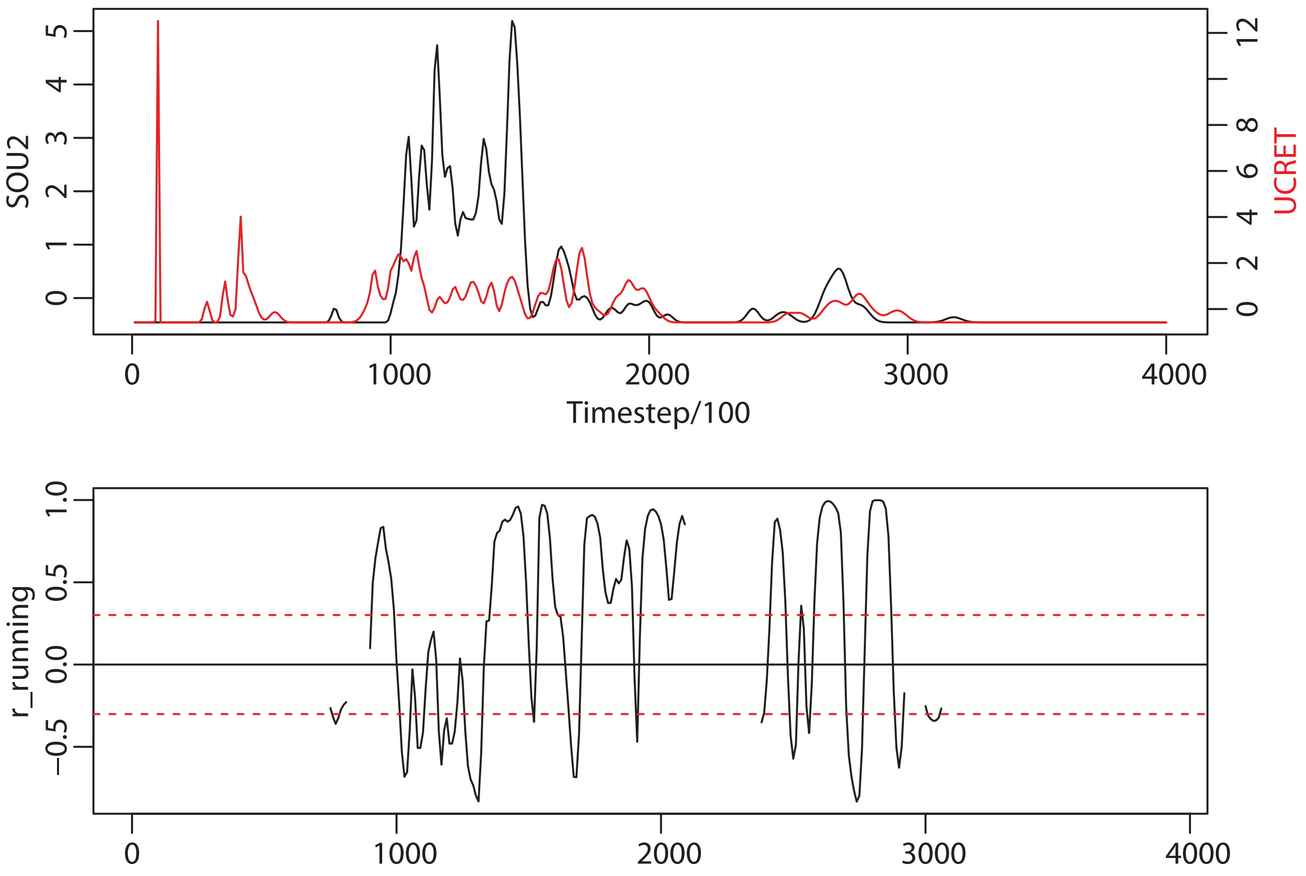

SOU2
UCRET
Timestep/100
r_running

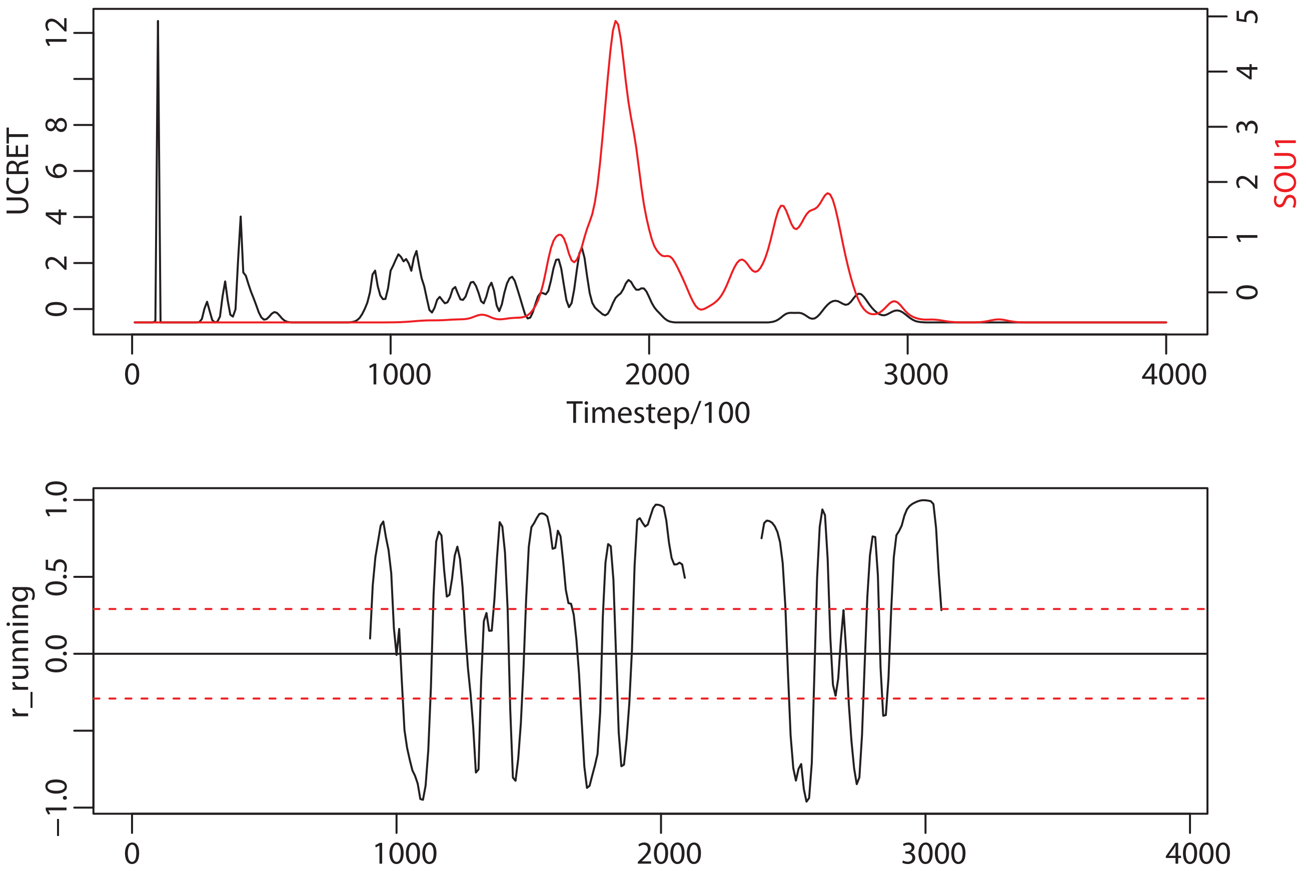

UCRET
SOU1
Timestep/100
r_running
0
1000
2000
3000
4000

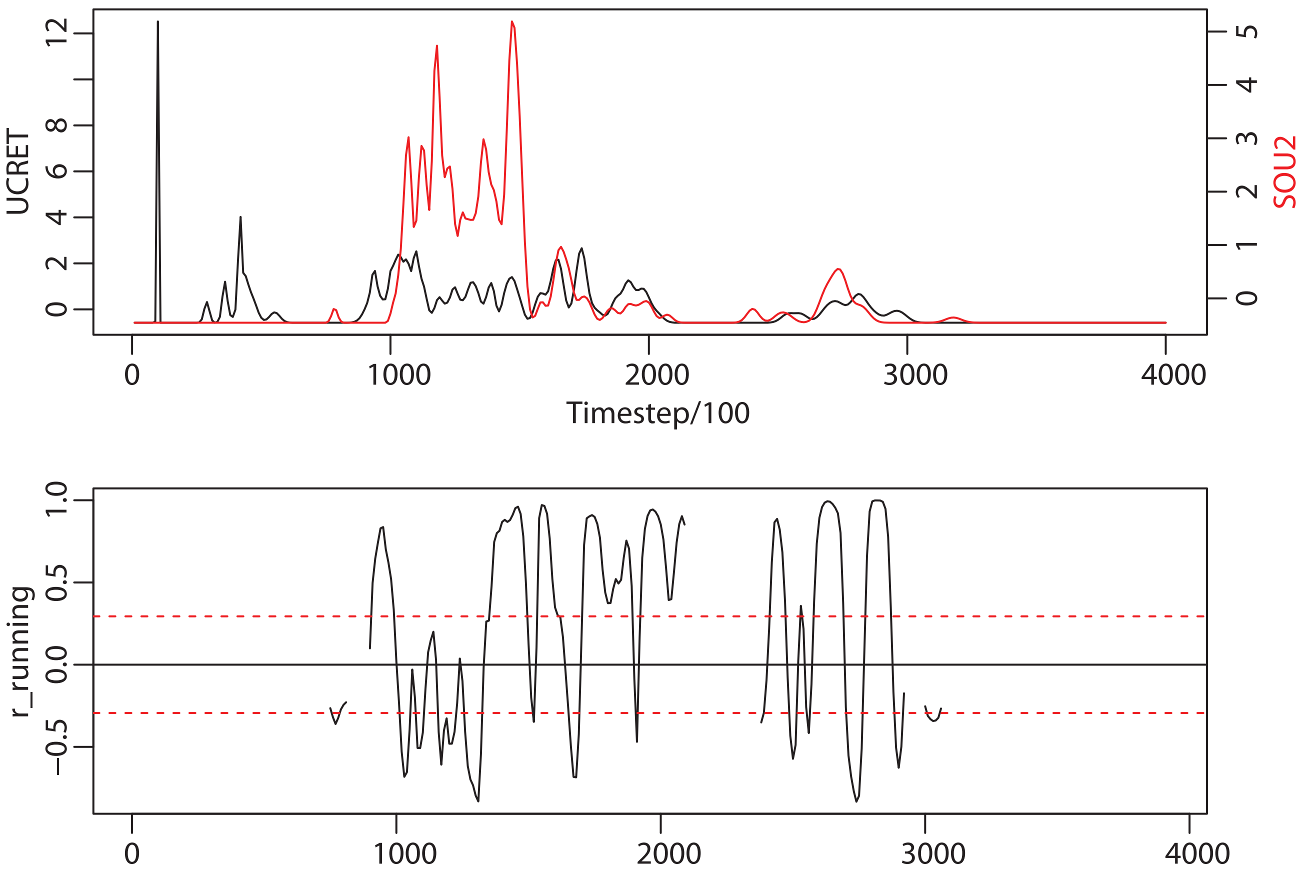
UCRET
SOU2
Timestep/100
r_running
0
2
4
6
8
12
0
1
2
3
4
5
0
1000
2000
3000
4000
−0.5
0.0
0.5
1.0

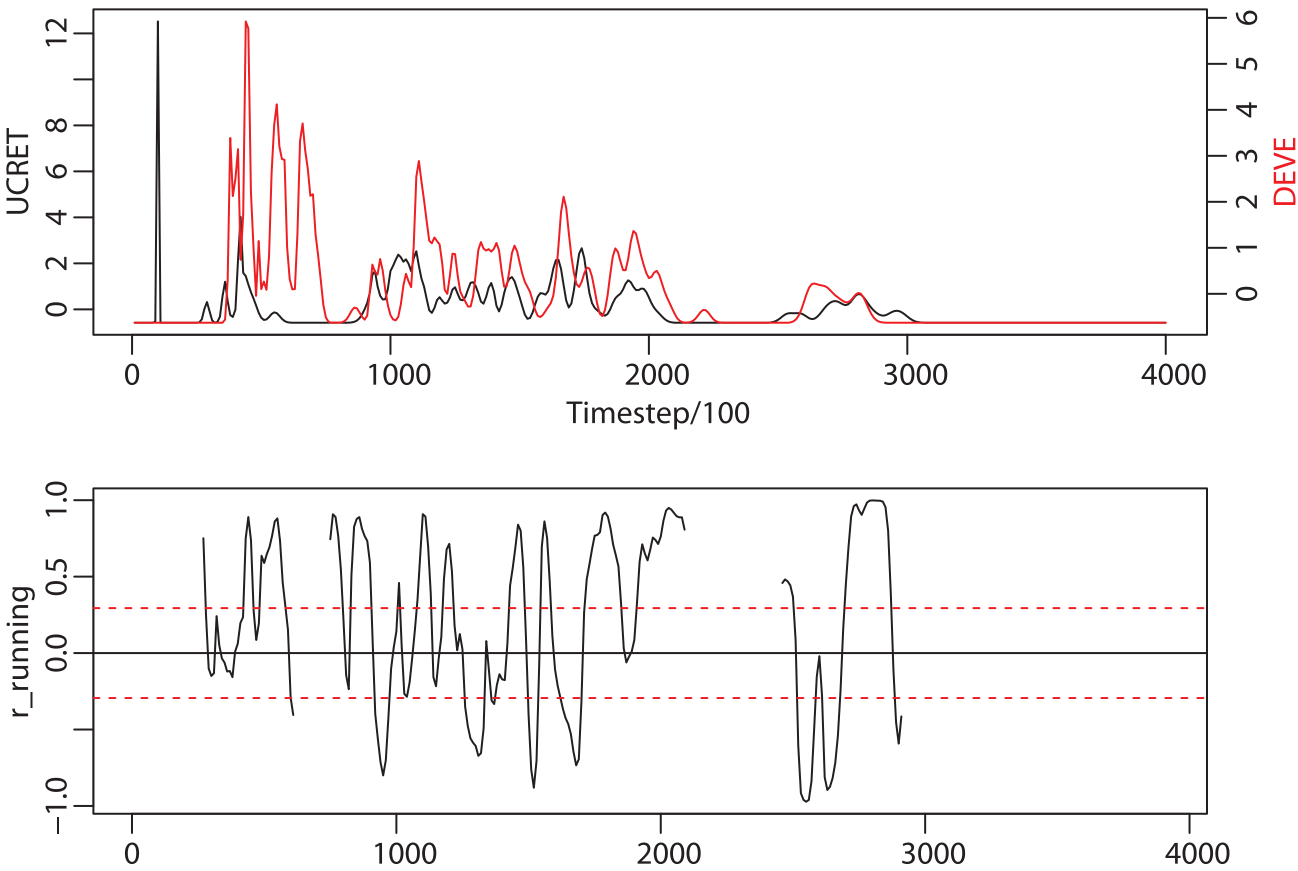
UCRET
DEVE
Timestep/100
r_running
0
2
4
6
8
12
1000
2000
3000
4000
−1.0
0.0
0.5
1.0

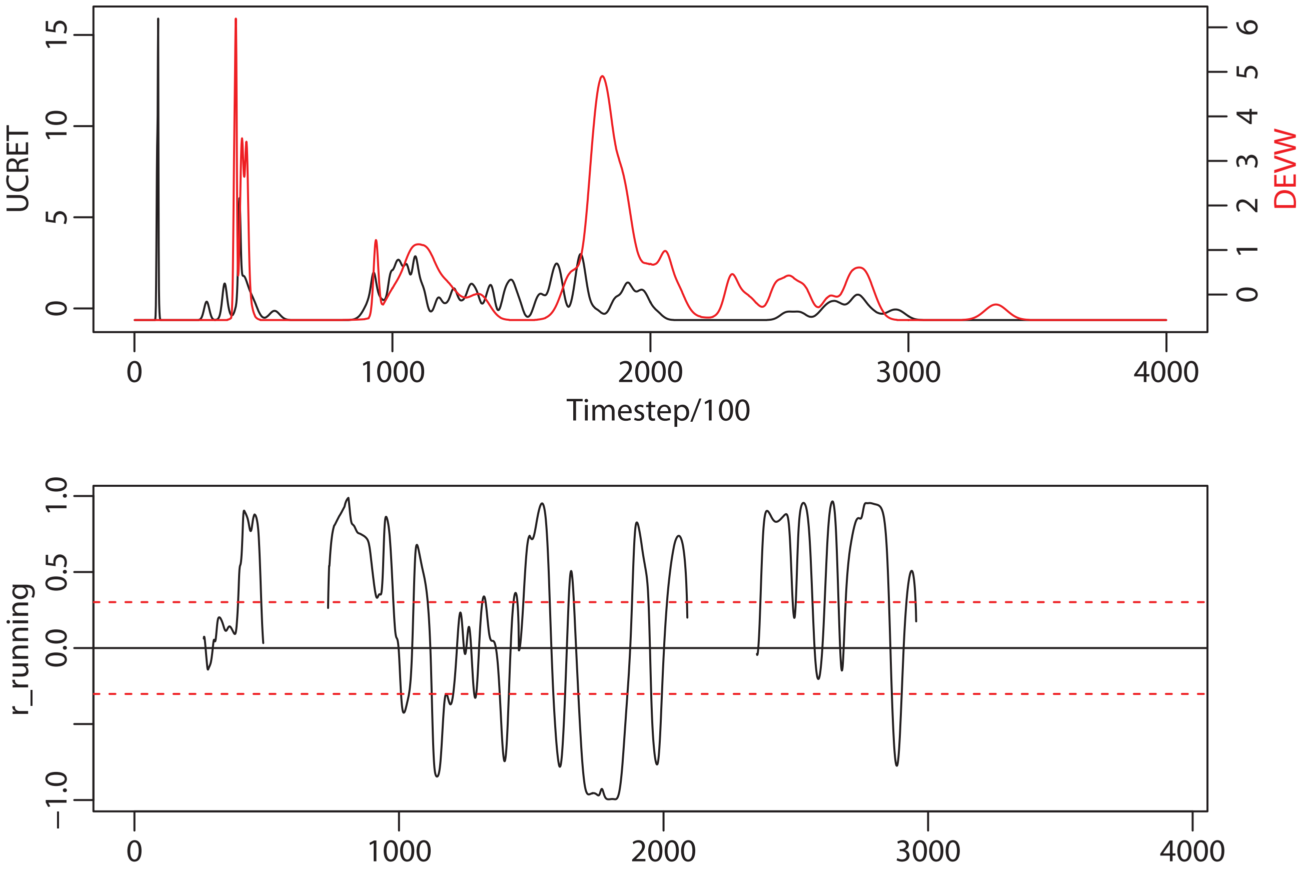

UCRET
DEWW
Timestep/100
r_running

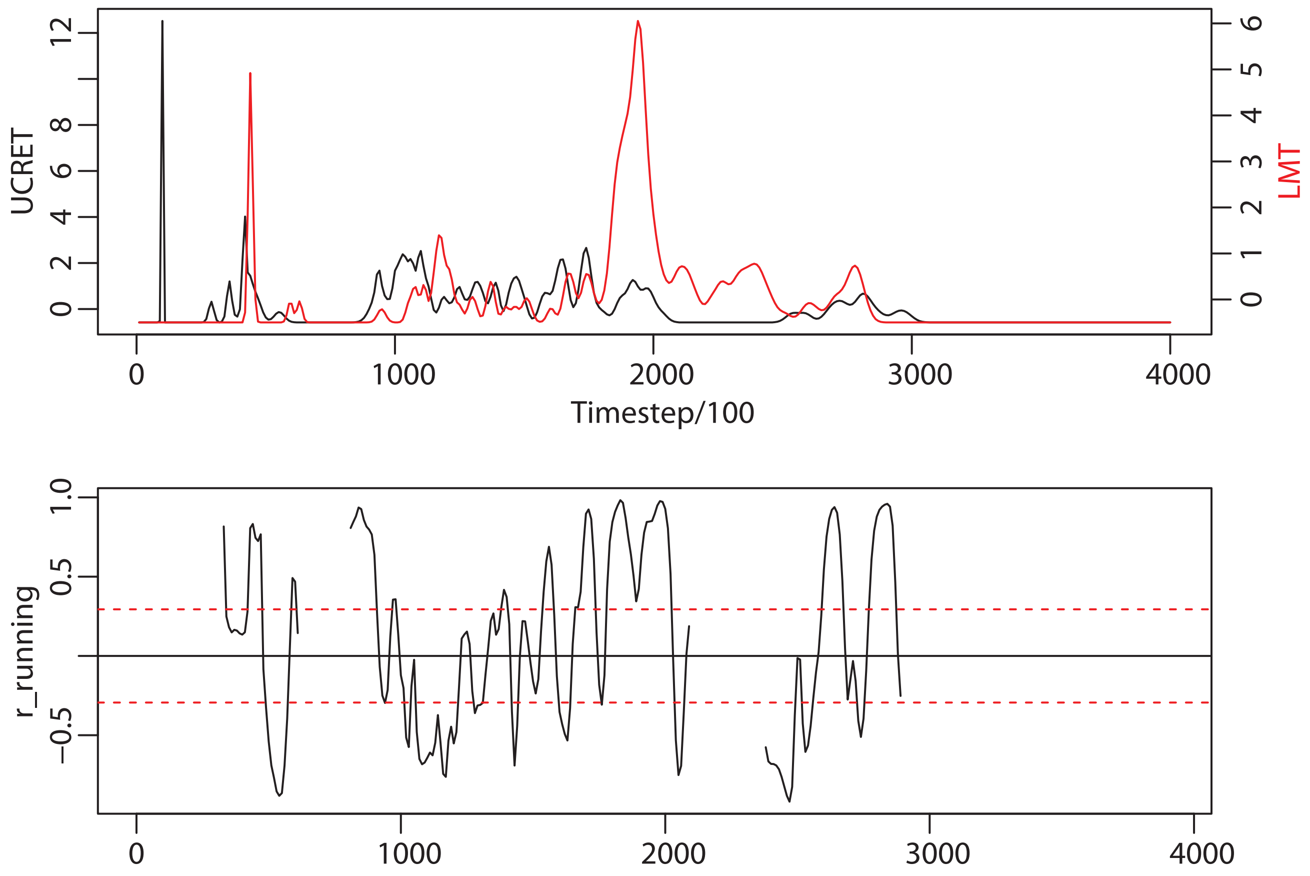
UCRET
LMT
Timestep/100
r_running
0
1000
2000
3000
4000
12
8
6
4
2
0
1
2
3
4
5
6
1.0
0.5
−0.5

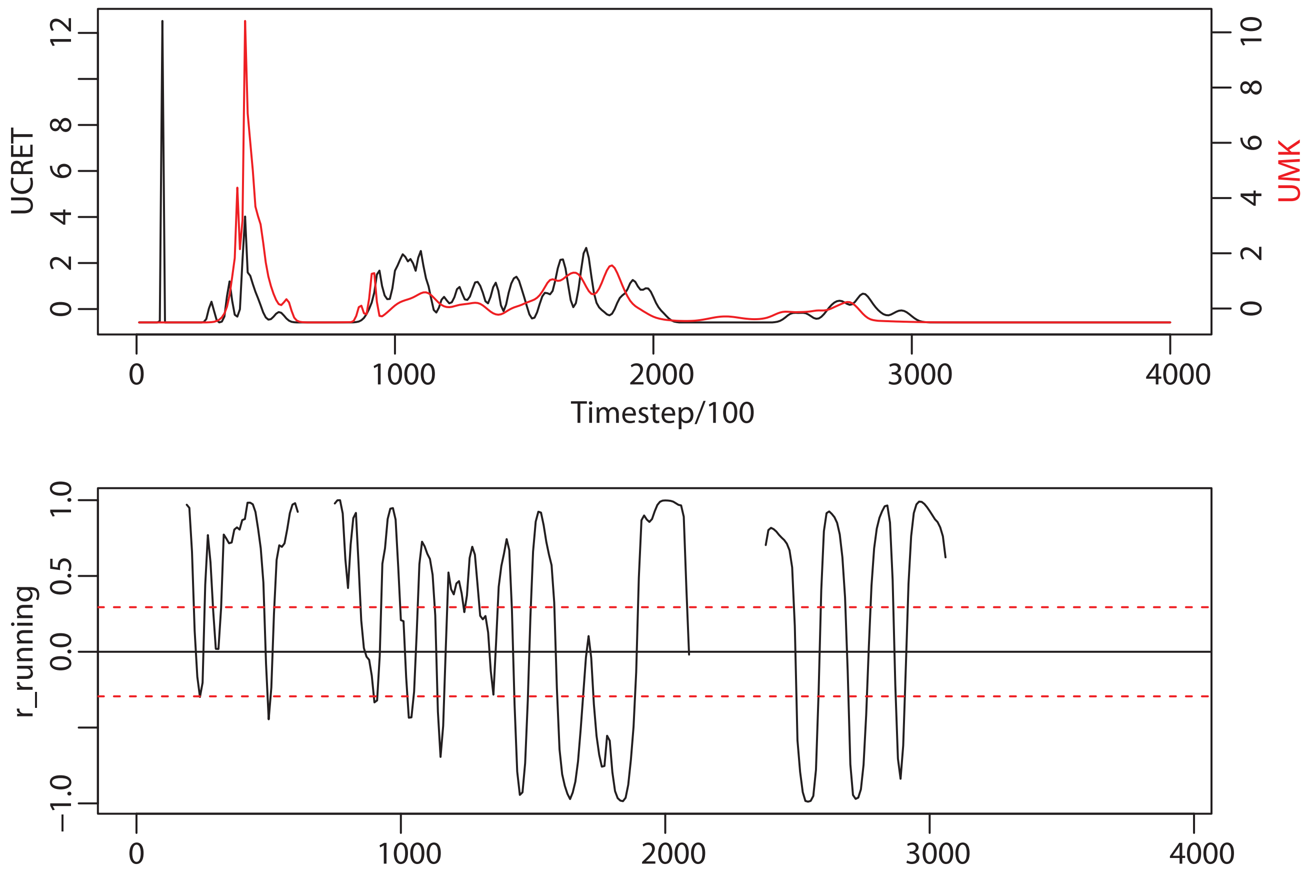

UCRET
UMK
Timestep/100
r_running

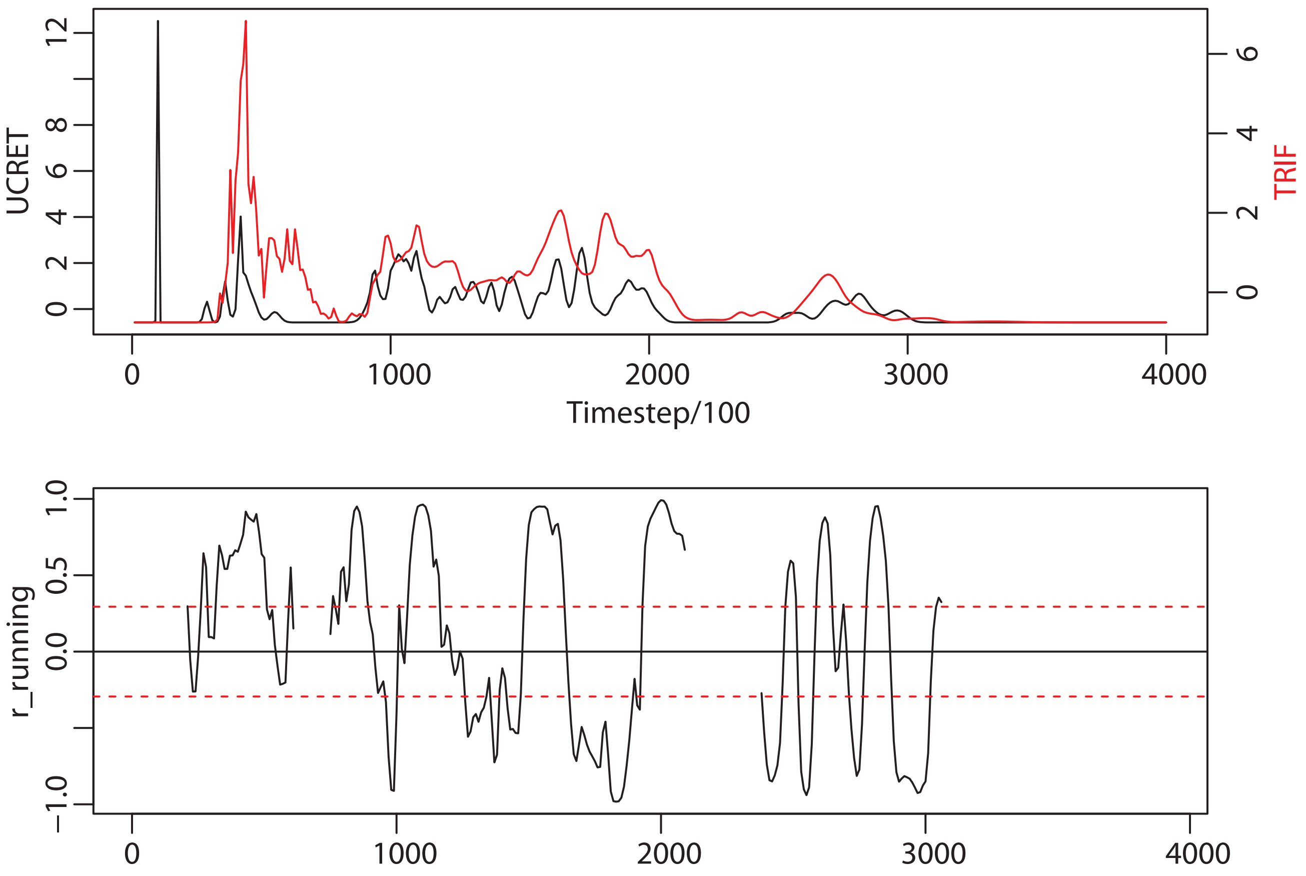

UCRET
TRIF
Timestep/100
r_running

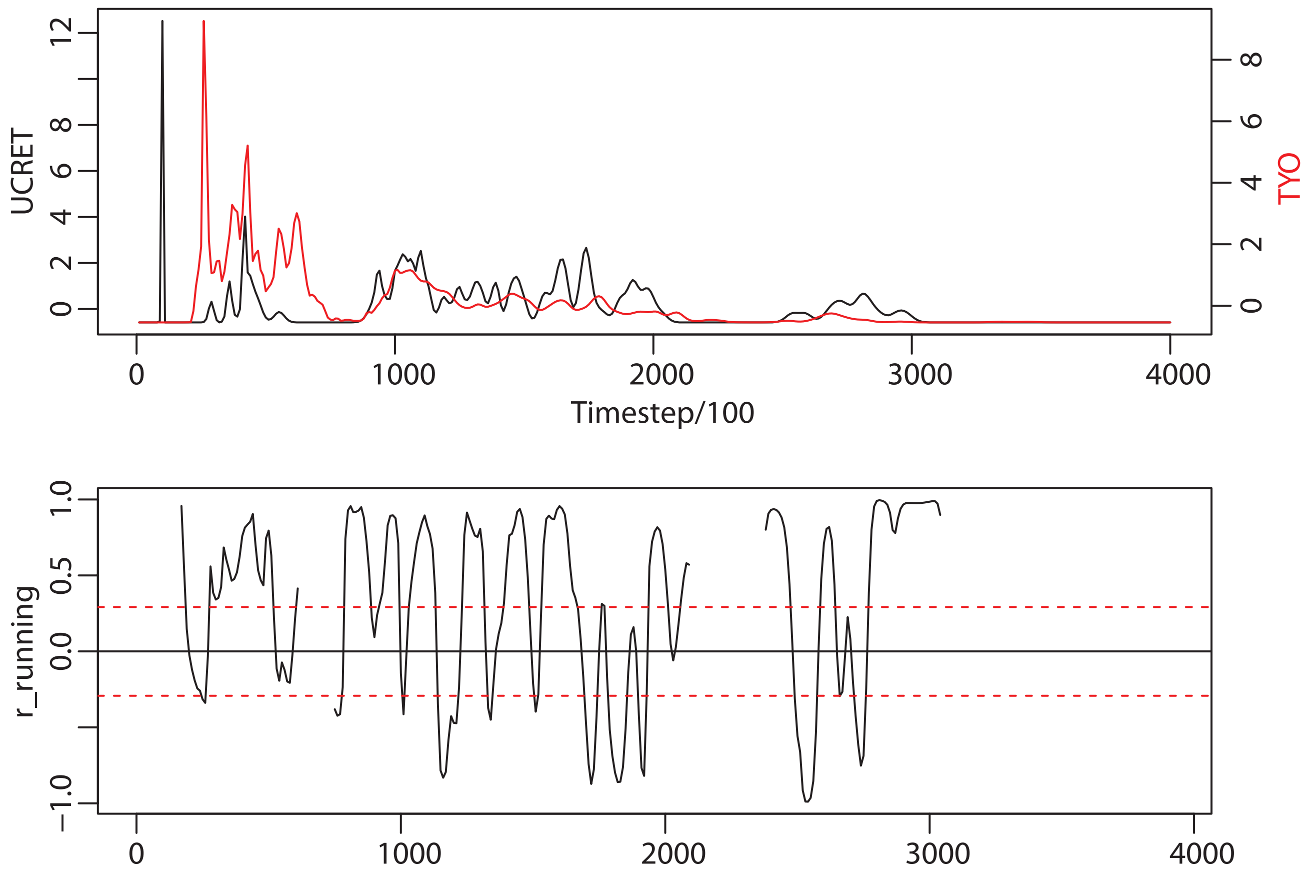
UCRET
TYO
Timestep/100
r_running
0
1000
2000
3000
4000
0
2
4
6
8
12
0.0
0.5
1.0
−1.0
