# Supplementary material for: 1.8 Billion Years of Detrital Zircon Recycling Calibrates a Refractory Part of Earth’s Sedimentary Cycle
Source: PLoS One. 2015 Dec 14;10(12):e0144727. doi: 10.1371/journal.pone.0144727 (PMC4682852; doi:10.1371/journal.pone.0144727)
Supplement: S1 Table — (PDF) [file pone.0144727.s006.pdf]

Table DR1: Data sources for Figs. 1, 2, 3, 4

Grouping	Number of ages (n) and samples (s)	References
Upper Cretaceous	s = 3, n = 101	This study, Hadlari et al. (2014)
Triassic overlap assemblage	s = 16, n=1108	Beranek and Mortensen (2011)
Triassic Interior Platform	s=9, n=520	Beranek et al. (2010b)
Upper Mississippian	s=3, n=118	Beranek et al. (2010a)
Lower Mississippian	s=2, n=173	Lemeiux et al. (2011)
Devonian East	s=3, n=161	Beranek et al. (2010a), Lemeiux et al. (2011)
Devonian West	s=2, n=61	Beranek et al. (2010a)
Supersource 2	s=7, n=242	Rainbird et al. (1997), Villeneuve et al. (1998), Hadlari et al. (2012)
Supersource 1	s=8, n=483	Piercey and Colpron (2009), Furlanetto et al. (2008), Lane and Gehrels (2014)
Figure 1: bedrock geology from Canada		Wheeler et al., (1996)
Figure 1: bedrock geology from Alaska		Beikman (1980), Plafker et al (1994)
Figure 1: topography		Can3D300 dem, http://Geogratis.ca
Figure 1: bathymetry		Jakobsson et al. (2008)
