# Supplementary material for: 1.8 Billion Years of Detrital Zircon Recycling Calibrates a Refractory Part of Earth’s Sedimentary Cycle
Source: PLoS One. 2015 Dec 14;10(12):e0144727. doi: 10.1371/journal.pone.0144727 (PMC4682852; doi:10.1371/journal.pone.0144727)
Supplement: S2 Table — (PDF) [file pone.0144727.s007.pdf]

S2 Table 2: Data sources for palynology

	Identification	GSC sample	Specimen #	Citation
Fig 5.1	*Densosporites* sp.	GSC Loc. C-562162	GSC 136 546	Bell (2015)
Fig 5.2	*Diatomozonotriletes* sp.	GSC Loc. C-562170	GSC 136 547	Bell (2015)
Fig 5.3	*Triquitrites* sp.	GSC Loc. C-562158	GSC 136 548	Bell (2015)
Fig 5.4	taeniate bisaccate pollen	GSC Loc. C-562161	GSC 136 549	Bell (2015)
